# Adjunctive Hyperbaric Oxygen Therapy for Severe Traumatic Limb Injuries: A Systematic Review and Meta-Analysis

**DOI:** 10.3390/medicina62081589

**Published:** 2026-08-18

**Authors:** Kang Kook Choi, Youngmin Kim, Dong Keon Yon, Wu Seong Kang

**Affiliations:** 1 Department of Traumatology, Gachon University College of Medicine, Incheon 21565, Republic of Korea; calvin511@naver.com; 2 Department of Trauma Surgery, Gachon University Gil Medical Center, Incheon 21565, Republic of Korea; skyofmay@gilhospital.com; 3 Center for Digital Health, Medical Science Research Institute, Kyung Hee University Medical Center, Kyung Hee University College of Medicine, Seoul 02447, Republic of Korea; 4 Department of Pediatrics, Kyung Hee University College of Medicine, Seoul 02447, Republic of Korea

**Keywords:** hyperbaric oxygenation, wound healing, wounds and injuries, soft tissue injuries, meta-analysis

## Abstract

*Background and Objectives*: Severe traumatic limb injuries, particularly those involving extensive soft tissue damage, present significant challenges in clinical management. Thus, we aimed to critically assess the effectiveness of hyperbaric oxygen therapy (HBOT) as an adjunctive treatment for severe traumatic limb injuries. *Materials and Methods*: For this systematic review and meta-analysis, we conducted a systematic search of MEDLINE/PubMed, Embase, and the Cochrane Library for studies published up to 7 March 2026. We included studies evaluating the effectiveness of adjunctive HBOT in patients with severe limb injuries. The prespecified primary outcome was complete wound healing, while secondary outcomes included time to wound healing, wound necrosis, length of hospital stay (LOS), and wound infection. Risk of bias was assessed using the ROBINS-I tool for observational studies and the Cochrane RoB 2.0 for randomized controlled trials (RCTs). *Results*: Seven studies (three RCTs and four observational) were included. A high risk of bias was identified in two RCTs, and a serious risk of bias was noted across all four observational studies. Complete wound healing was reported in one randomized trial and favored HBOT, but the certainty of evidence was very low. The time to wound healing did not differ between the HBOT and control groups (MD, −1.44; 95% CI, −6.70 to 3.82, I^2^ = 0%). The LOS did not differ between the HBOT and control groups (MD, −3.39; 95% CI, −14.74 to 7.79, I^2^ = 94%). However, wound infection rates were lower in the HBOT group compared to the control group (OR, 0.31; 95% CI, 0.22–0.45, I^2^ = 20%). Similarly, wound necrosis rates were reduced in the HBOT group (OR, 0.23; 95% CI, 0.12–0.45, I^2^ = 41%). *Conclusions*: Evidence is insufficient to determine whether HBOT improves complete wound healing. Although HBOT may reduce wound infection and necrosis, the certainty of evidence was low to very low.

## 1. Introduction

Severe traumatic limb injuries, especially those involving extensive soft tissue damage, present significant challenges in clinical management [1,2,3,4]. These injuries are often complicated by a high incidence of infections, tissue necrosis, and delayed wound healing, which can significantly extend recovery times and increase morbidity. While conventional treatments, such as surgical intervention and meticulous wound care, are foundational, they often do not fully address the complexities of these injuries.

Hyperbaric oxygen therapy (HBOT) has been increasingly explored as a supplemental treatment in the management of traumatic injuries [3,5,6,7,8]. HBOT involves the delivery of 100% oxygen at elevated atmospheric pressures, typically within a hyperbaric chamber, which enhances oxygen delivery to hypoxic tissues [9]. This process is believed to facilitate wound healing by improving oxygenation of damaged tissues, reducing edema through vasoconstriction, and modulating inflammatory responses [9]. Additionally, HBOT may mitigate the harmful effects of ischemia-reperfusion injury, which is a common consequence of severe limb trauma [9].

However, the role of HBOT in treating severe traumatic limb injuries remains under investigation, with studies reporting mixed results [3,5,6,7,8]. While some research indicates significant benefits in terms of accelerated wound healing and reduced complications, other studies have not demonstrated consistent advantages [3,8]. These discrepancies may stem from variations in study design, patient characteristics, or HBOT protocols, highlighting the need for a comprehensive evaluation of the available evidence.

This systematic review and meta-analysis aimed to critically assess the effectiveness of hyperbaric oxygen therapy (HBOT) as an adjunctive treatment for severe traumatic limb injuries. Furthermore, recently published studies not included in prior systematic reviews [3,5,8], which searched the literature up to November 2022, were incorporated into the present analysis. By systematically analyzing the available evidence, we aimed to determine whether HBOT improves clinical outcomes, including wound healing and infection rates, and to evaluate its potential role in standard trauma care.

## 2. Materials and Methods

### 2.1. Published Study Search and Selection Criteria

This study was conducted in accordance with the Preferred Reporting Items for Systematic Reviews and Meta-Analyses (PRISMA) guidelines [10]. (Supplementary Table S1) The study protocol was prospectively registered in PROSPERO (CRD42024564685; https://www.crd.york.ac.uk/prospero accessed on 22 May 2026). Relevant articles were obtained through comprehensive searches of the MEDLINE/PubMed, Embase, and Cochrane databases for the period up to 7 March 2026. Relevant search strategies were employed to explore these databases, as detailed in Supplementary Table S2. An additional manual search was performed by scrutinizing the reference lists of pertinent articles. All retrieved articles underwent meticulous screening of titles and abstracts to assess their eligibility for inclusion. Review articles and meta-analyses were also examined to identify any supplementary studies that met the eligibility criteria. Finally, the search results were thoroughly reviewed, and articles that investigated HBOT in patients with severe soft tissue injuries in the extremities were included.

The prespecified primary outcome was complete wound healing, defined as the proportion of patients achieving complete wound healing according to the definition used in each primary study. Secondary outcomes included time to wound healing, wound necrosis, wound infection, length of hospital stay, other complications, and mortality. Wound infection was considered a specific wound-related complication within the category of “other complications” prespecified in the PROSPERO registration. Outcomes expressed as the proportion of patients achieving complete healing and those expressed as time to complete healing were analyzed separately because they represented conceptually and statistically distinct outcome measures. Wound-healing outcomes were classified according to their conceptual definition and data type. Complete wound healing was defined as the proportion of patients achieving complete healing according to the definition used in each primary study. Time to wound healing included only outcomes reporting an elapsed time to complete or clinically defined wound healing. Time to granulation formation, serial wound-size reduction, wound-depth reduction, granulation scores, and composite wound-assessment scores were not considered equivalent to time to complete healing and were not pooled with time-to-healing outcomes. Because no uniform definition was used across the primary studies, study-specific definitions were retained and reported transparently. The inclusion criteria for this review were as follows: (i) trauma patients with severe soft tissue injuries in the extremities; (ii) patients who underwent HBOT; (iii) a comparison between HBOT and control with conventional treatment; (iv) availability of pertinent outcomes; and (v) provision of odds ratios (ORs), means with standard deviations, or data enabling their calculation. Both randomized controlled trials (RCTs) and observational studies were included. Studies examining diseases other than the specified conditions, non-original research articles, and publications in languages other than English were excluded from the analysis.

### 2.2. Data Extraction

Data extraction was performed by two investigators (K.K.C. and W.S.K.). The following key information was extracted from each study: first author’s name, year of publication, study location, study design, study period, number of patients included in the analysis, patient age, location of injury, injury severity, method of HBOT, concurrent surgical treatment, rate of wound healing, time to wound healing, length of hospital stay, and complications. HBOT treatment was defined as the protocolized provision of inhaled oxygen in a hyperbaric chamber during the early stage of hospitalization. Treatments that only targeted local tissue with hyperbaric oxygen were excluded. Discrepancies were resolved by consultation with a third reviewer (D.K.Y.).

### 2.3. Quality Assessment

To evaluate the risk of bias, we utilized a revised tool for assessing risk of bias in RCTs (RoB 2.0) [11] for randomized controlled trials and the Risk of Bias in Non-randomized Studies of Interventions (ROBINS-I) tool [12] for observational studies. All studies were independently reviewed by two investigators. Any disagreements concerning study selection and data extraction were resolved through consensus.

### 2.4. Statistical Analysis

Statistical analyses were conducted using RevMan software version 5.4 (Review Manager; The Cochrane Collaboration, 2020). We performed meta-analyses using ORs for binary outcomes and mean differences (MDs) for continuous outcomes. Confidence intervals (CIs) were determined using exact confidence limits for binomial proportions. ORs were pooled using the Mantel-Haenszel method, while MDs were calculated using the inverse variance method [13]. Heterogeneity was visually evaluated via forest plots and quantitatively assessed using I^2^ statistics and Cochran’s Q test, with *p*-values less than 0.10 deemed statistically significant [13]. The degree of heterogeneity was interpreted based on I^2^, where 25%, 50%, and 75% were predefined as thresholds for low, moderate, and high inconsistency, respectively [14]. If significant heterogeneity was detected (I^2^ > 50%), a random-effects model was applied. Due to a limited number of studies (less than 10), it was not feasible to assess publication bias using methods like funnel plots and Egger regression tests [15,16]. Because substantial clinical heterogeneity was anticipated, randomized and non-randomized studies were analyzed separately. Additional subgroup analyses according to injury mechanism, anatomical site, or HBOT protocol were considered but were not undertaken when fewer than two studies were available within a clinically comparable subgroup. Leave-one-out sensitivity analyses were performed to evaluate the influence of individual studies with large statistical weight or clinically distinct populations. Because the review focused on severe traumatic limb injuries, the >50 cm^2^ wound-area subgroup reported by Chang et al. [17] was selected post hoc for the main analysis of the relevant outcomes, whereas the ≤50 cm^2^ subgroup was examined in a sensitivity analysis. A two-sided *p* value of less than 0.05 was considered statistically significant.

### 2.5. Certainty of Evidence

The certainty of evidence for each clinically relevant outcome was assessed using the Grading of Recommendations Assessment, Development and Evaluation (GRADE) approach. Two reviewers (K.K.C. and W.S.K.) independently evaluated the certainty of evidence for each outcome using the GRADE approach [18]. Any discrepancies in the domain judgments or overall certainty ratings were resolved through discussion. A third reviewer adjudicated unresolved disagreements (D.K.Y.). The evaluated outcomes were complete wound healing, time to wound healing, length of hospital stay, wound infection, and wound necrosis. Because randomized controlled trials and non-randomized studies have different initial certainty ratings and methodological limitations, certainty was assessed separately for each study-design subgroup using the corresponding pooled estimate. Evidence from randomized trials initially received a high-certainty rating, whereas evidence from non-randomized studies initially received a low-certainty rating. Certainty was downgraded by one or two levels for serious or very serious concerns, respectively, in the domains of risk of bias, inconsistency, indirectness, imprecision, and publication bias. Risk-of-bias judgments were informed by RoB 2 for randomized trials and ROBINS-I for non-randomized studies. Inconsistency was evaluated using the direction and magnitude of study effects, overlap of confidence intervals, and statistical heterogeneity. Indirectness was assessed according to the applicability of the population, intervention, comparator, and outcome. Imprecision was judged based on sample size, number of events, confidence-interval width, and inclusion of clinically different effects. Potential upgrading factors for non-randomized evidence were considered, but no upgrades were applied. Summary of Findings tables were generated using GRADEpro GDT [19].

### 2.6. Protocol Deviations and Amendments

The review generally followed the prespecified PROSPERO protocol. The search was updated through 7 March 2026. Complete wound healing was prespecified as the primary outcome in the registered PROSPERO protocol (CRD42024564685) and remained the primary outcome in this review. However, it was summarized narratively because only one study reported a comparable dichotomous endpoint. Wound infection was analyzed separately within the prespecified category of other complications. The post hoc selection of the >50 cm^2^ subgroup from Chang et al. [17] for the main analysis was based on clinical applicability to the review population and was evaluated through a sensitivity analysis using the ≤50 cm^2^ subgroup. Study-design subgroup analyses were performed as planned, whereas anatomical-site subgroup analyses were not feasible because most subgroups contained only one study. GRADE assessments and additional sensitivity analyses were added during revision to evaluate the certainty and robustness of the findings.

## 3. Results

### 3.1. Selection and Characteristics

**Figure 1:**
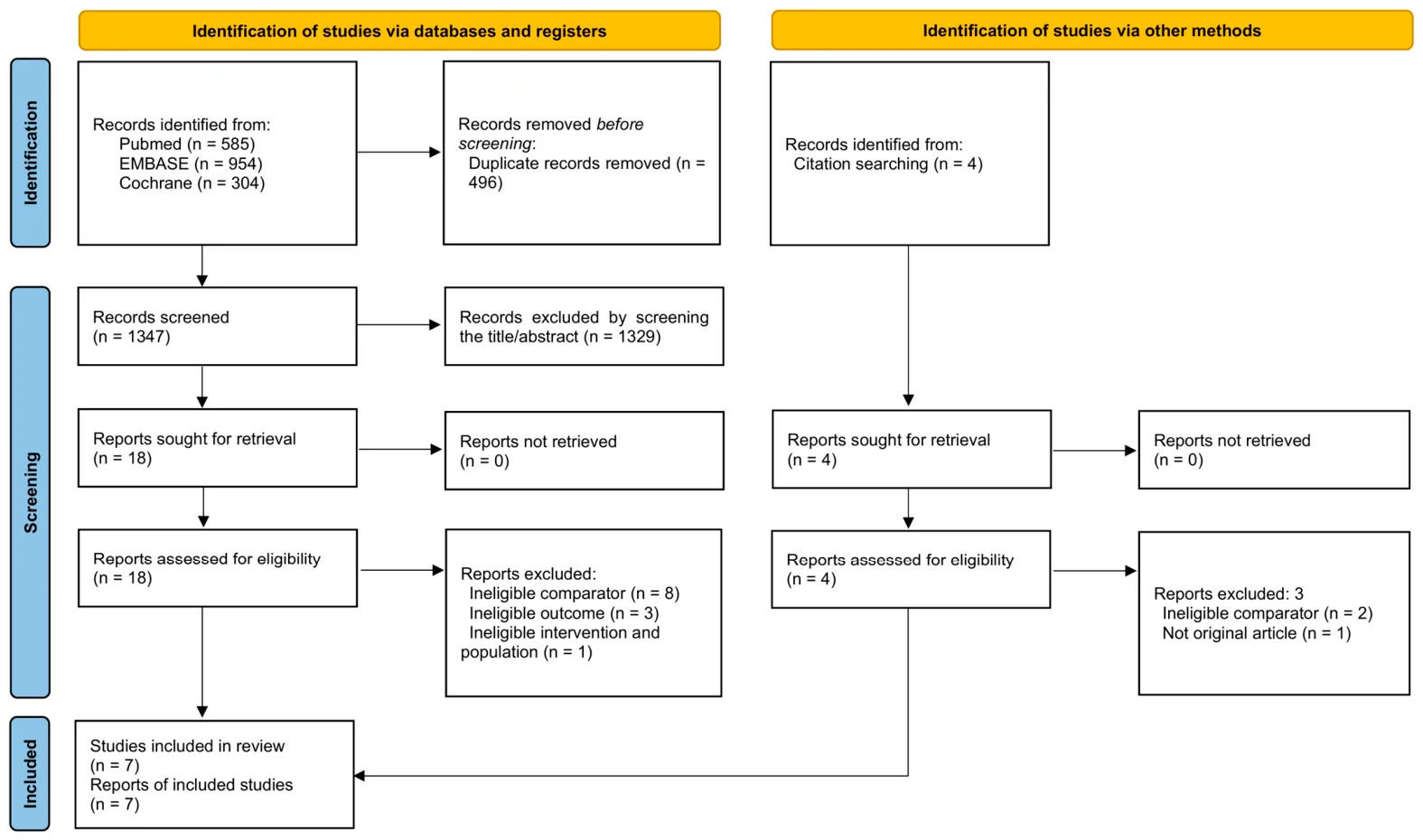
Flowchart of study selection.

A systematic search identified 1347 studies after duplicate records had been removed using a citation manager. Of these, 1329 studies were excluded following title and abstract screening. Consequently, 18 full-text articles were assessed for eligibility, of which 12 were excluded for the following reasons: ineligible comparator (k = 8), ineligible outcome (k = 3), and ineligible intervention (k = 1). In addition, four studies were identified through citation searching of previous systematic review [8]. Among these, three studies were excluded because they were ineligible comparator (k = 2) or non-original articles (k = 1). Ultimately, seven studies [17,20,21,22,23,24,25] comprising 762 patients met the predetermined eligibility criteria and were included in this systematic review and meta-analysis (Figure 1).

### 3.2. Included Studies

Detailed information on the eligible studies is summarized in Table 1. Three studies [20,23,24] were RCTs, and four [17,21,22,25] were observational studies. One study [23] was multicenter trial whereas other studies were conducted at single center. Among the RCTs, Millar et al. [23] conducted the primary analysis on an unadjusted intention-to-treat basis. Because six patients assigned to the control group subsequently received HBOT, the investigators also performed a separate as-per-treatment-received analysis, which did not identify statistically significant differences between the treatment groups. For the present meta-analysis, we used the results reported according to the original randomized allocation. The other two RCTs [20,24] reported no crossover and analyzed participants according to their assigned groups. The study by Millar et al. [23] is the only multicenter trial, having recruited patients across 10 international hospitals. The other six studies [17,20,21,22,24,25] were all conducted at single centers. The trial by Millar et al. [23] randomized 60 patients to each group and conducted its primary analysis on an unadjusted intention-to-treat basis. However, two patients assigned to HBOT withdrew and declined follow-up, and one patient assigned to the control group had insufficient data for meaningful analysis. Consequently, primary outcome data were available for 58 patients assigned to HBOT and 59 patients assigned to the control group, all of whom were analyzed according to their original randomized allocation. Because six patients assigned to the control group subsequently received one or more HBOT sessions, the investigators also performed a separate as-per-treatment-received analysis. The present meta-analysis, however, used the results based on the original randomized allocation. The study by Roje et al. [21] was the only one to specifically investigate war-related injuries, focusing on casualties from the 1991–1995 conflicts in Croatia and Bosnia. In contrast, the remaining six studies [17,20,22,23,24,25] exclusively evaluated civilian trauma, such as motor vehicle accidents, occupational crush injuries, and falls. Regarding the anatomical distribution of injuries, the focus varied significantly across the included literature. Marra et al. [25] exclusively evaluated soft tissue trauma distal to the knee (the leg and foot), while Millar et al. [23] focused specifically on open tibial fractures. Similarly, Chang et al. [17] restricted their analysis to crush injuries of the hand. In contrast, Bouachour et al. [20], Roje et al. [21], and Yamada et al. [22] included a combination of upper and lower extremity injuries (e.g., arm, hand, and forearm/leg). Finally, Raj et al. [24] evaluated general open musculoskeletal trauma without limiting inclusion to a specific limb or anatomical region. The concurrent or adjunctive use of Negative Pressure Wound Therapy (NPWT) alongside HBOT was explicitly documented in only two studies. [17,25] The remaining five studies [20,21,22,23,24] did not report the use of NPWT, relying instead on conventional wound management.

### 3.3. Excluded Studies

Following full-text assessment, 14 studies [26,27,28,29,30,31,32,33,34,35,36,37,38,39] were excluded because they did not meet the prespecified eligibility criteria (Table 2). The principal reasons were the absence of an eligible comparator or insufficient comparative data, including non-comparative study designs (k = 10), an ineligible intervention or population (k = 1), and an ineligible outcome (k = 3). No study was excluded solely on the basis of statistical or clinical heterogeneity identified during data synthesis. Specifically, Butler et al. [28] evaluated an ineligible intervention and population, Ince et al. [36] focused on peripheral nerve repair rather than wound-related clinical outcomes, and Oley et al. [34] assessed vascular endothelial growth factor protein and mRNA expression rather than eligible clinical outcomes.

### 3.4. Risk of Bias Assessment of Included Studies

**Figure 2:**
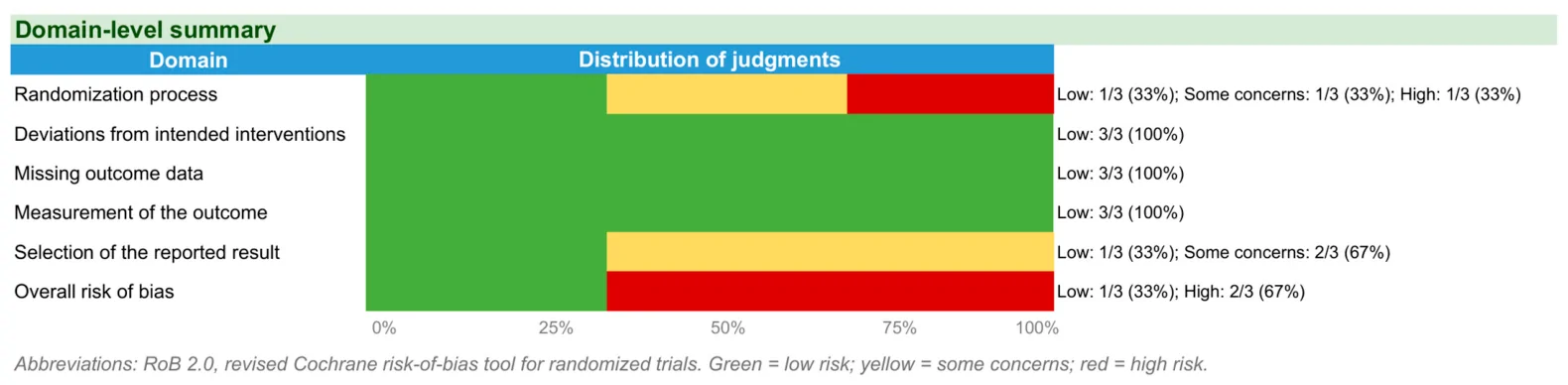
Domain-level summary of RoB 2.0.

Regarding the randomized studies (Table 3 and Figure 2), the risk of bias varied significantly across domains. Only Millar et al. [23] was judged to have a low overall risk of bias, demonstrating a robust study design. In contrast, Bouachour et al. [20] and Raj et al. [24] were classified as having a high overall risk of bias. Specifically, Bouachour et al. [20] provided insufficient details regarding the randomization process, and Raj et al. [24] lacked information on allocation concealment and pre-specified protocols, leading to concerns in the selection of reported results.

**Figure 3:**
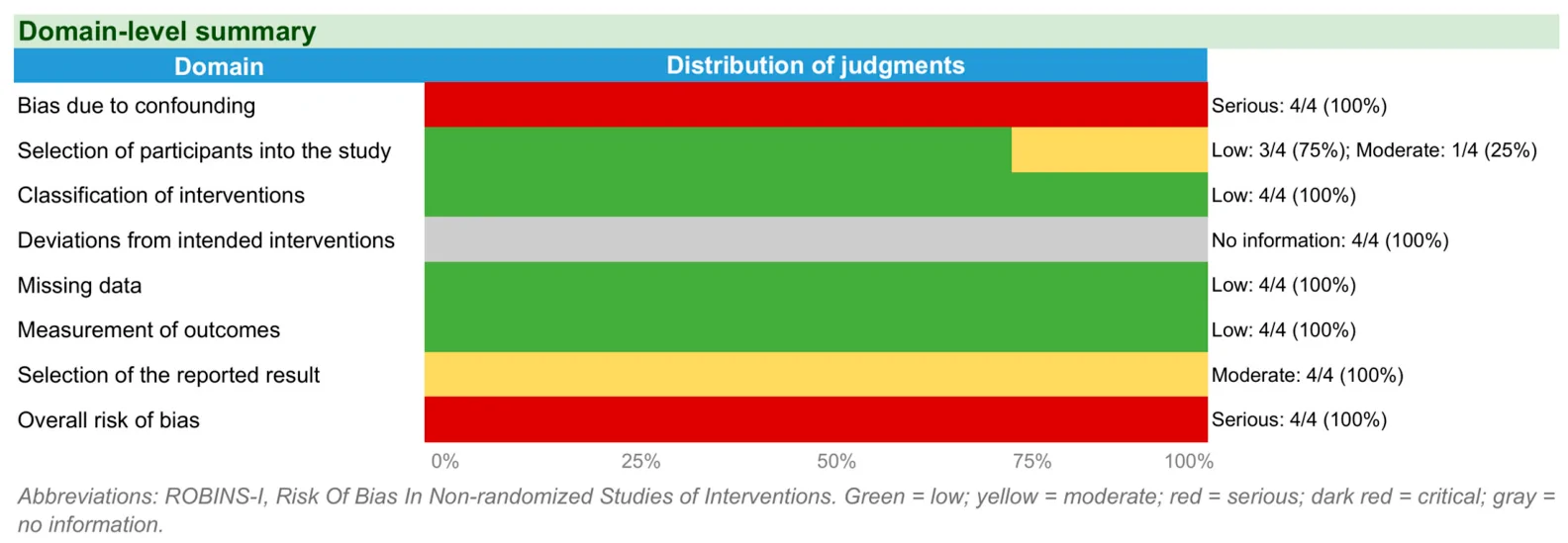
Domain-level summary of ROBINS-I.

For the non-randomized studies (Table 4 and Figure 3), all four included papers [17,21,22,25] were assessed as having a serious overall risk of bias. Most studies failed to adequately account for baseline clinical differences between groups. For instance, Yamada et al. [22] utilized a historical control with a limited sample size, while Chang et al. [17] and Marra et al. [25] reported significant baseline imbalances, such as differences in initial wound surface area or patient characteristics. Additionally, “moderate” risks were noted in the selection of reported results due to the lack of prospective registered protocols for these observational cohorts.

### 3.5. Meta-Analysis of Clinical Outcomes: HBOT vs. Control Group

Complete wound healing, the prespecified primary outcome, was reported as a dichotomous outcome in one study. Bouachour et al. [20] defined complete wound healing as healing without tissue necrosis requiring surgical excision. Complete healing was achieved in 17 of 18 patients in the HBOT group and 10 of 18 patients in the placebo group (*p* = 0.009). Because only one study reported this outcome in a comparable dichotomous form, a meta-analysis was not performed.

For Chang et al. [17], outcome data were reported separately for wound areas > 50 cm^2^ and ≤50 cm^2^. Because the review focused on severe traumatic limb injuries, the >50 cm^2^ subgroup was used in the primary analyses. The ≤50 cm^2^ subgroup was evaluated in sensitivity analyses. The two subgroup estimates were not entered simultaneously into the same meta-analysis.

**Figure 4:**
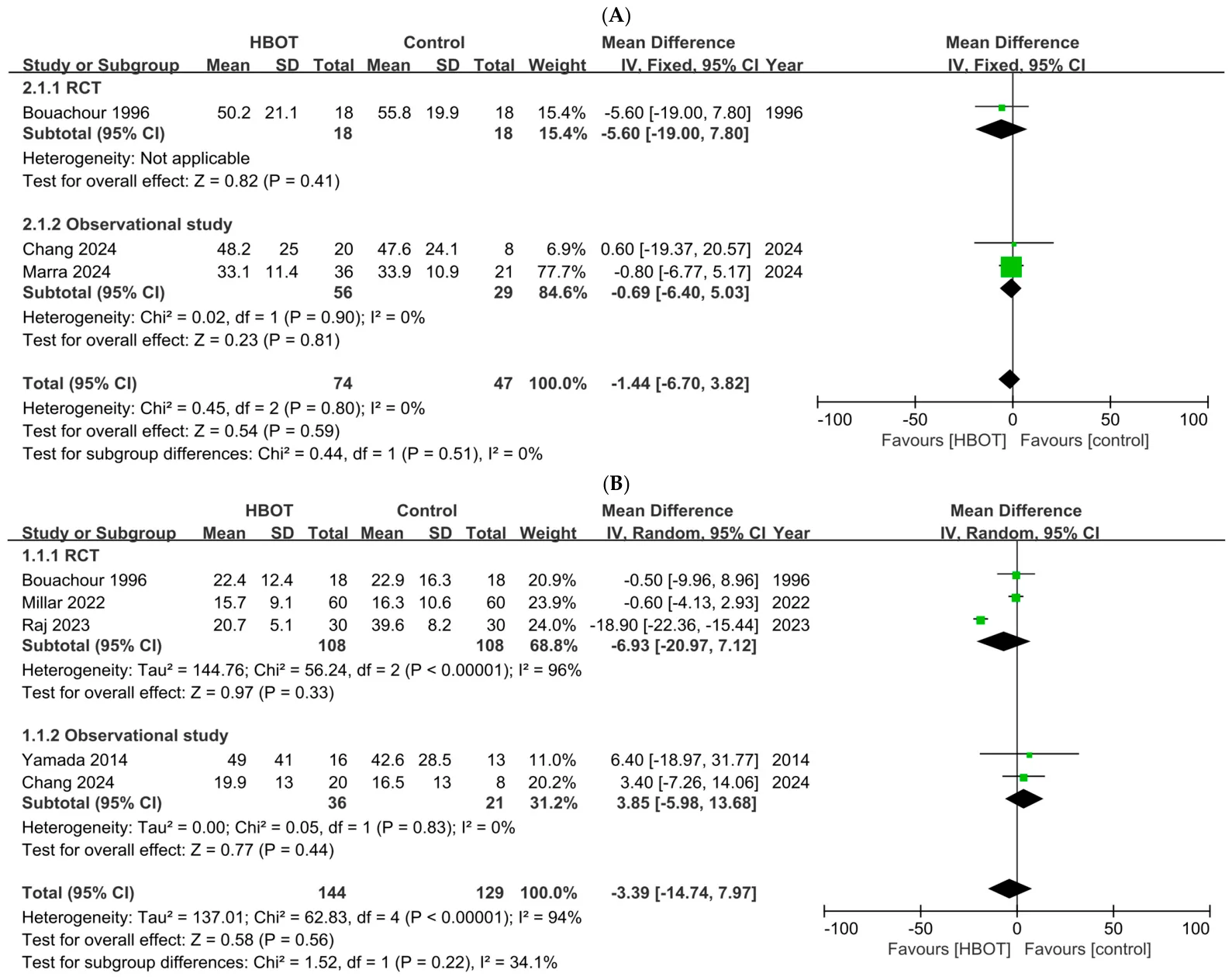

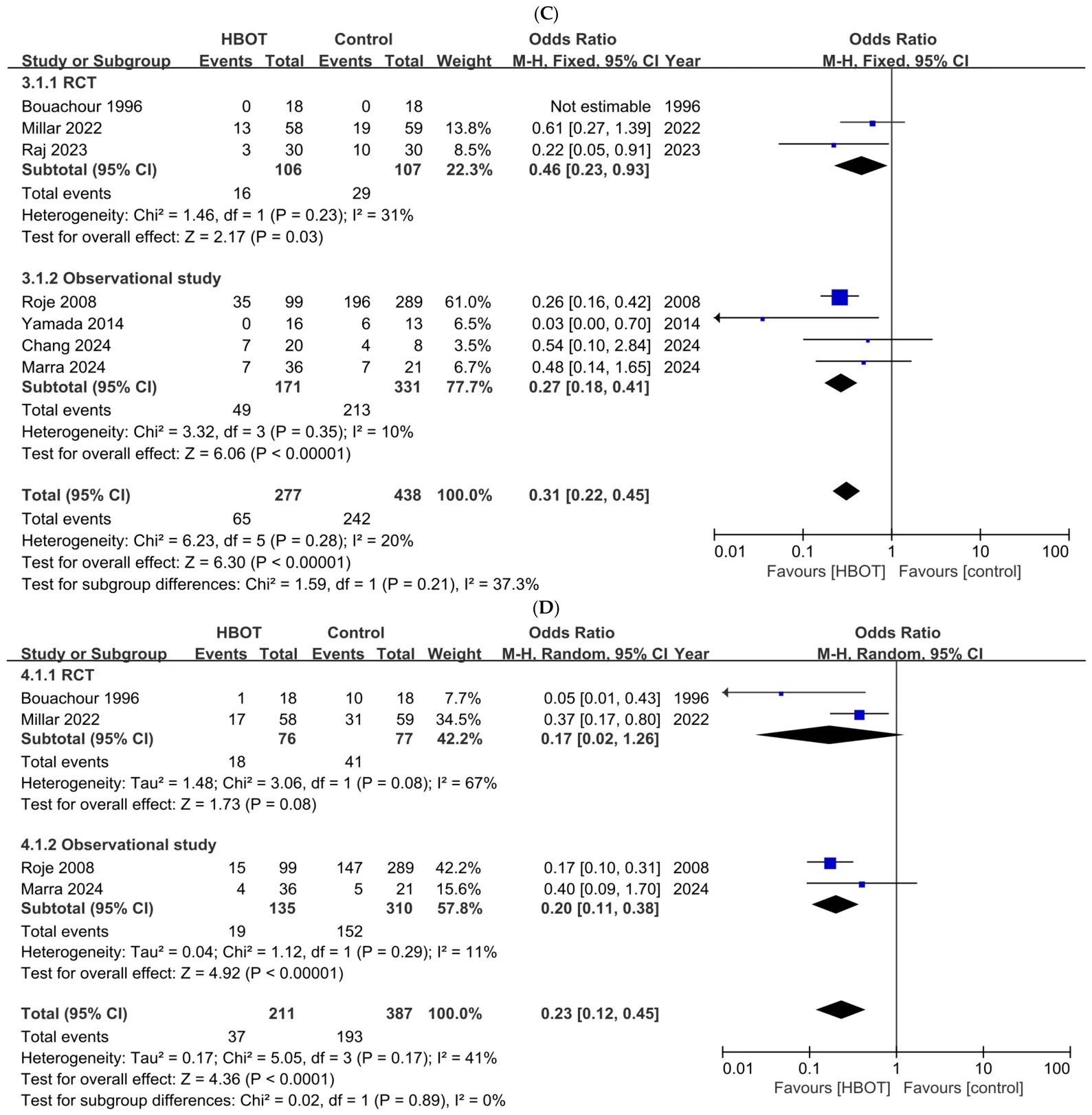
Forest plot in terms of (**A**) time to wound healing (days) (Bouachour 1996 [20], Chang 2024 [17], Marra 2024 [25]), (**B**) length of hospital stay (days) (Bouachour 1996 [20], Millar 2022 [23], Raj 2023 [24], Yamada 2014 [22], Chang 2024 [17]), (**C**) wound infection (Bouachour 1996 [20], Millar 2022 [23], Raj 2023 [24], Roje 2008 [21], Yamada 2014 [22], Chang 2024 [17], Marra 2024 [25]), and (**D**) wound necrosis (Bouachour 1996 [20], Millar 2022 [23], Roje 2008 [21], Marra 2024 [25]).

The pooled analysis showed no significant difference between the HBOT group and the control group regarding the time to wound healing (Figure 4A). The overall MD was −1.44 days (95% CI: −6.70 to 3.82; *p* = 0.59), with no observed heterogeneity (I^2^ = 0). Subgroup analysis also revealed no significant improvements in either the RCT (*p* = 0.41) or observational study (*p* = 0.81) categories. Similarly, the length of hospital stay did not differ significantly between the two groups (Figure 4B). The total MD was −3.39 days (95% CI, −14.74 to 7.97; *p* = 0.56). Notably, high statistical heterogeneity was observed for this outcome (I^2^ = 94%), primarily driven by the RCT subgroup (I^2^ = 96%), where Raj et al. [24] reported a significantly shorter stay while other studies showed negligible differences. In contrast to the recovery duration, HBOT demonstrated a significant protective effect against wound-related complications (Figure 4C). The risk of wound infection was significantly lower in the HBOT group compared to the control group (OR, 0.31; 95% CI, 0.22 to 0.45; *p* < 0.00001), with low heterogeneity (I^2^ = 20%). This benefit was consistent across both RCTs (OR, 0.46; 95% CI, 0.23 to 0.93; *p* = 0.03) and observational studies (OR, 0.27; 95% CI, 0.18 to 0.41; *p* < 0.00001). The overall pooled analysis favored HBOT for wound necrosis (OR, 0.23; 95% CI, 0.12–0.45; *p* < 0.0001) (Figure 4D). In the observational subgroup, HBOT was associated with lower odds of wound necrosis (OR, 0.20; 95% CI, 0.11–0.38; *p* < 0.00001). However, this result was largely driven by Roje et al. [21], which had the largest sample size and was judged to be at serious risk of bias. In the RCT subgroup, the pooled estimate favored HBOT but did not reach statistical significance (OR, 0.17; 95% CI, 0.02–1.26; *p* = 0.08; I^2^ = 67%).

Among the included literature, multivariate analyses performed by Roje et al. [21] and Millar et al. [23] demonstrate that adjusted analyses suggested associations after accounting for selected measured covariates; however, residual confounding and imprecision remain. Roje et al. [21] found that after adjusting for the initial emergency surgical strategy, the absence of adjuvant HBOT remained a significant and independent risk factor for deep soft-tissue infection, flap necrosis, skin graft lysis, and osteomyelitis. Similarly, utilizing mixed-effects logistic regression, Millar et al. [23] revealed that HBOT significantly decreased the odds of acute tissue necrosis (adjusted odds ratio [OR] 0.28) and the combined outcome of infection and necrosis (adjusted OR 0.16), even after controlling for injury severity and multicenter variables. However, the adjusted estimates were not pooled because only a limited number of studies reported adjusted ORs and the covariates included in the models were not sufficiently comparable across studies.

### 3.6. Sensitivity Analysis

To evaluate the robustness of our meta-analysis findings and identify potential sources of heterogeneity, a leave-one-out sensitivity analysis was performed by sequentially omitting each study and recalculating the pooled estimates for all outcomes (Supplementary Figures S1–S4). Sensitivity analysis for the time to wound healing confirmed the stability of the initial non-significant result. The pooled MD fluctuated minimally and remained statistically non-significant across all exclusions, indicating that the overall estimate was not driven by any individual study (Supplementary Figure S1). The sensitivity analysis for the length of hospital stay yielded a critical finding regarding statistical heterogeneity. While the overall effect remained non-significant in most iterations, the exclusion of Raj et al. [24] resulted in a dramatic reduction in heterogeneity, with the I^2^ value dropping from 94% to 0% (Supplementary Figure S2C). This identifies Raj et al. [24] as the primary source of the high heterogeneity observed in the length of hospital stay outcome. Despite this, the overall conclusion of no significant difference between the HBOT and control groups remained consistent across all sensitivity iterations. The significant protective effect of HBOT against wound infection and wound necrosis remained highly robust. For wound infection, the pooled odds ratio (OR) remained consistently significant across all iterations, ranging from 0.22 to 0.46, without any single study significantly altering the overall direction or statistical significance (*p* < 0.05 for all) (Supplementary Figure S3). Similarly, the reduction in wound necrosis remained stable, with the pooled OR consistently favoring HBOT regardless of which study was excluded (Supplementary Figure S4). Because Roje et al. [21] contributed a large proportion of the observational events and weight and included a clinically distinct war-related trauma population, additional attention was given to analyses excluding this study. For wound infection, exclusion of Roje et al. [21] attenuated but did not eliminate the association favoring HBOT (overall OR, 0.40; 95% CI, 0.23–0.69; I^2^ = 9%, Supplementary Figure S3). The corresponding observational estimate remained statistically significant (OR, 0.32; 95% CI, 0.13–0.76; I^2^ = 31%). For wound necrosis, the overall estimate also remained significant after exclusion of Roje et al. [21] (OR, 0.28; 95% CI, 0.11–0.73; I^2^ = 37%, Supplementary Figure S4). However, the observational subgroup was then represented by only one study and was not statistically significant (OR, 0.40; 95% CI, 0.09–1.70). These findings suggest that the overall direction of effect was not solely determined by Roje et al., although the magnitude and certainty of the pooled estimates remained sensitive to the available study composition.

Sensitivity analyses using the ≤50 cm^2^ subgroup from Chang et al. are presented in Supplementary Figure S5. For time to wound healing, the pooled estimate among observational studies remained non-significant but favored HBOT (MD, −3.74 days; 95% CI, −8.79 to 1.30; I^2^ = 69%), and the overall pooled estimate was MD −3.97 days (95% CI, −8.69 to 0.75; I^2^ = 40%). For wound infection, the observational pooled estimate remained significant in favor of HBOT (OR, 0.29; 95% CI, 0.19–0.45; I^2^ = 52%), and the overall pooled OR was 0.33 (95% CI, 0.23–0.47; I^2^ = 43%). These findings did not materially alter the overall interpretation of the primary analyses.

### 3.7. Certainty of Evidence

The certainty of evidence ranged from low to very low across the evaluated outcomes (Table 5). Complete wound healing was supported by very-low-certainty evidence from one randomized trial because of serious risk of bias and very serious imprecision. For time to wound healing, both randomized and non-randomized evidence were rated as very low certainty. The randomized evidence was downgraded for serious risk of bias and very serious imprecision, whereas the non-randomized evidence was downgraded for serious risk of bias and serious imprecision. Evidence for length of hospital stay was also rated as very low certainty in both study-design subgroups. Randomized evidence was limited by serious risk of bias, very serious inconsistency, and serious imprecision, while non-randomized evidence was limited by serious risk of bias and serious imprecision. Randomized evidence for wound infection was rated as low certainty because of serious risk of bias and serious imprecision, although the pooled estimate favored HBOT. Non-randomized evidence for wound infection was rated as very low certainty because of serious confounding concerns. For wound necrosis, randomized evidence was rated as very low certainty because of serious risk of bias, inconsistency, and very serious imprecision. Non-randomized evidence was also rated as very low certainty because of serious risk of bias, despite low heterogeneity and a comparatively precise effect estimate favoring HBOT.

## 4. Discussion

### 4.1. Major Findings

The prespecified primary outcome of complete wound healing was reported as a dichotomous outcome in only one small randomized trial. Although a higher proportion of patients achieved complete healing with HBOT, the certainty of evidence was very low, and the finding could not be quantitatively pooled. Therefore, the current evidence remains insufficient to establish whether HBOT improves the probability of complete wound healing in patients with severe traumatic limb injuries. In the pooled analyses of secondary outcomes, HBOT was associated with lower incidences of wound infection and wound necrosis, whereas no clear differences were observed in time to wound healing or length of hospital stay. However, the certainty of evidence for these outcomes was low to very low because of risk of bias, imprecision, clinical heterogeneity, and outcome-specific statistical inconsistency. Accordingly, the observed associations should be interpreted cautiously and should not be regarded as definitive evidence of clinical benefit.

### 4.2. Comparison with Previous Research

Previous systematic reviews investigating the effectiveness of HBOT in patients with trauma have yielded promising yet cautious conclusions. Several reviews [3,5,6,7,8] indicate that adjunctive HBOT may be beneficial for acute traumatic injuries, particularly crush injuries and compromised skin grafts. For instance, Wang et al. [5] and Eskes et al. [3] found that HBOT can improve complete wound healing, enhance the survival of split-skin grafts, and reduce the incidence of tissue necrosis and the need for additional surgical procedures. More recently, Kwee et al. [8] found that incorporating HBOT into standard trauma care for severe lower limb soft tissue injuries holds the potential to positively influence wound healing and reduce both necrosis and infection rates. Despite these favorable clinical outcomes, the overall quality of the existing evidence remains a significant limitation. Reviews consistently highlight that available studies are often hampered by methodological flaws, inadequate controls, and small sample sizes. Furthermore, Bennett et al. [7] concluded that there is currently insufficient evidence from randomized trials to definitively support or refute the use of HBOT for acute fracture healing or established non-union. While HBOT is generally considered safe, severe adverse events such as barotrauma and oxygen toxicity seizures have occasionally been reported, necessitating careful patient selection. Consequently, while previous systematic reviews advocate for the potential clinical benefits of HBOT in trauma management, they emphasize the need for larger, high-quality RCTs to establish standardized protocols. This context informed our current systematic review and meta-analysis, which identified a limited number of well-designed RCTs and substantial heterogeneity. The present meta-analysis provides a quantitative summary of the available comparative evidence but does not establish a definitive treatment effect.

### 4.3. Clinical Implication of Present Study

HBOT has been investigated as a potential adjunct to standard treatment in patients with crush injuries, compartment syndrome, and acute traumatic ischemia. In the present review, pooled estimates suggested possible reductions in wound infection and tissue necrosis. However, the low to very low certainty of evidence precludes firm conclusions regarding its effectiveness or recommendations for its routine use. Decisions regarding HBOT should therefore be individualized and integrated with timely surgical management, vascular assessment, infection control, and standard trauma care. Large, multicenter randomized controlled trials are required to determine whether the observed associations represent clinically meaningful treatment effects.

Importantly, two of the included studies performed adjusted analyses to account for major clinical confounders. Roje et al. [21] used multiple logistic regression and included the initial emergency surgical strategy, specifically adherence to NATO emergency war surgery recommendations, as a covariate. After adjustment, the absence of adjunctive HBOT remained independently associated with an increased risk of deep soft-tissue infection, flap necrosis, skin graft lysis, and osteomyelitis. Similarly, Millar et al. [23] applied a mixed-effects logistic regression model that incorporated Gustilo grade, severe wound contamination, and muscle loss as fixed effects, with the recruiting center included as a random effect. HBOT was associated with lower odds of acute tissue necrosis (adjusted OR, 0.28) and the composite outcome of infection and necrosis (adjusted OR, 0.16). Although residual confounding cannot be excluded, these adjusted findings suggest that the observed benefits of HBOT may not be solely attributable to differences in baseline injury severity or treatment setting. Rather, adjunctive HBOT may provide an independent clinical benefit in reducing selected wound complications, particularly tissue necrosis and infection, in patients with severe traumatic limb injuries. Although these adjusted analyses were consistent with the direction of the pooled findings, residual confounding cannot be excluded, and an independent treatment effect of HBOT cannot be established. Therefore, these findings should be regarded as supportive evidence rather than conclusive evidence.

The pooled estimates for wound infection and wound necrosis were strongly influenced by Roje et al. [21], the largest observational study, which involved war-related Gustilo type III injuries and had serious concerns regarding confounding. This population differed clinically from the predominantly civilian crush-injury and open-fracture cohorts included in the other studies. Considerable variability was also present in HBOT protocols, including treatment pressures ranging from approximately 1.0 to 2.8 ATA and treatment courses ranging from 2 to approximately 25 sessions. Accordingly, the pooled estimates should not be interpreted as representing a uniform treatment effect across all forms of severe limb trauma. Nevertheless, leave-one-out analyses excluding Roje et al. [21] showed that the overall associations for wound infection and wound necrosis remained in favor of HBOT. The infection estimate remained statistically significant, whereas the observational necrosis estimate became non-significant and was based on only one remaining study. These findings reduce, but do not eliminate, concern that the observed benefits were driven by a single study. Given the clinical heterogeneity and low or very low certainty of evidence, these results should be regarded as hypothesis-generating.

Regarding safety, major HBOT-related complications were uncommon in the included studies. Millar et al. [23] reported no major complications, although treatment was discontinued in two patients because of minor ear barotrauma and in several others because of nausea, vomiting, pain, agitation, or anxiety. Chang et al. [17] and Marra et al. [25] reported no HBOT-related complications. Nevertheless, adverse events were not systematically or consistently reported across all studies, precluding a quantitative safety analysis. Therefore, the apparent tolerability of HBOT should be interpreted cautiously.

### 4.4. Future Research Direction

HBOT in our review involved both monoplace and multiplace chambers, each presenting distinct clinical trade-offs. Monoplace chambers offer logistical advantages in terms of space efficiency and cost-effectiveness; however, they preclude direct physical access to the patient during compression, thereby limiting their utility for the critically ill. Conversely, multiplace chambers facilitate concurrent treatment of multiple patients and allow medical staff to perform hands-on intensive care, including real-time hemodynamic monitoring and emergency interventions such as suction or defibrillation. In the context of polytrauma, the management of ICU patients necessitates specialized, hyperbaric-compatible equipment—specifically ventilators and infusion pumps engineered to maintain precision under fluctuating atmospheric pressures.

Future research should focus on three primary pillars. First, the adoption of biomarker-guided precision medicine—utilizing transcutaneous oximetry or molecular signatures such as vascular endothelial growth factor [34]—may optimize patient selection and outcomes. Second, the integration of artificial intelligence for real-time anomaly detection and the development of remotely controlled hyperbaric infrastructure represent critical technological priorities. Finally, large-scale multicenter trials are strictly warranted to standardize treatment protocols, defining optimal dosing regimens and the requisite number of sessions tailored to specific injury phenotypes.

### 4.5. Limitations

Our study has several limitations. First, the number of studies included in our analysis was limited, with only three RCTs available. Second, substantial clinical heterogeneity existed across the included studies with respect to injury mechanism, anatomical site, severity, treatment setting, and concomitant surgical procedures. The study populations included civilian and war-related trauma, crush injuries, open fractures, hand injuries, and lower-extremity soft-tissue injuries. HBOT protocols also varied considerably in treatment pressure, session duration, number of sessions, timing of initiation, and chamber type. Moreover, although two studies utilized negative-pressure wound therapy (NPWT), the potential effects of different wound-management modalities, including NPWT, and other adjunctive surgical approaches were not taken into account. These differences may have modified the observed treatment effects and limited the clinical interpretability and generalizability of a single pooled estimate. Although randomized and observational studies were analyzed separately and leave-one-out sensitivity analyses were performed, further subgroup analyses according to injury type, anatomical site, or HBOT regimen were not feasible because most clinically relevant subgroups contained only one study. Accordingly, the pooled estimates should be interpreted cautiously because they represent average effects across clinically heterogeneous populations and treatment protocols. Third, two RCTs were judged to be at high risk of bias. Furthermore, four observational studies were deemed to have serious concerns, primarily in the domain of confounding. The apparent reductions in wound infection and wound necrosis were driven largely by non-randomized studies, all of which had serious concerns regarding confounding and baseline imbalances. Therefore, residual confounding may explain part of the observed treatment effect. The randomized evidence generally favored HBOT but remained limited by small sample sizes, risk of bias, and imprecision. Accordingly, these findings should be interpreted as suggestive rather than definitive. Further well-designed RCTs are warranted. Fourth, publication bias was not formally assessed because of the limited number of included studies. Therefore, the possibility of small-study effects cannot be excluded. Positive studies of HBOT may have been more likely to be published, while null or unfavorable studies may have remained unpublished. In addition, several excluded studies reported favorable outcomes without eligible control groups, suggesting that the published literature may be enriched for positive uncontrolled evidence. Therefore, the observed pooled benefits may have been overestimated. Fifth, wound-healing outcomes were defined and measured inconsistently across studies. Complete healing, time to healing, time to granulation, wound-size reduction, and composite wound-assessment scores were not interchangeable, which limited the number of studies eligible for each quantitative synthesis. Finally, we excluded articles with non-English language, which may introduce language bias.

## 5. Conclusions

The available evidence is insufficient to determine whether adjunctive HBOT improves complete wound healing in patients with severe traumatic limb injuries because the prespecified primary outcome was reported in only one small trial. Although our meta-analysis suggests that HBOT may be associated with favorable secondary outcomes, including lower incidences of wound necrosis and wound infection, these findings were derived from clinically heterogeneous populations and HBOT protocols and were supported by low- or very-low-certainty evidence. Further well-designed randomized controlled trials are needed to clarify the clinical effectiveness of adjunctive HBOT.

## Figures and Tables

**Table 1:** Characteristics of eligible studies.

Author	Year	Study Design	Location	Study Period (Year)	Patient	Intervention (HBOT)	Intervention (Surgery)	Comparison	Outcomes
Bouachour et al. [20]	1996	RCT, single center	France	ND, 48 months	36 patients with crush injury of limbs, Gustilo type II or III	HBOT in multiplace hyperbaric chamber by inhaling 100% oxygen, 2.5 ATA, twice daily, over 6 days	Primary closure, stabilization procedure (internal, external, or conservative), vascular reconstruction, fasciotomies, and muscle rotation flap	HBOT (18) vs. control (placebo) (18)	necrosis, complete wound healing, repetitive surgical procedure, time to healing, serious infection, LOS
Roje et al. [21]	2008	observational, single center	Croatia	1991 to 1995	388 patients with complex war injuries in upper and lower extremity, Gustilo type III	HBOT by inhaling 100% oxygen in hyperbaric chamber, 1.0 to 2.8 ATA	NATO surgical strategy	HBOT (99) vs. control (289)	infection, necrosis, time to granulation
Yamada et al. [22]	2014	observational, single center	Japan	2004 to 2012	29 patients with crushing injuries with severities greater than Gustilo IIIA	HBOT, 2.0 ATA for 60 min daily, monoplace chamber, decided duration by discussion (2 to 12 session)	Emergency surgery performed within six hours of injury, for debridement and external fixation, secondary skin and muscle flap and graft if needed	HBOT (n = 16, 2008 to 2012) vs. control (n = 13, 2004 to 2008)	infection, additional surgical drainage or debridement procedures, ICU stay, LOS
Millar et al. [23]	2022	RCT, multicenter (10 hospitals)	Australia, Sweden, the Czech Republic, Portugal, Chile, Italy, Austria, India and the United States.	2007 to 2014	120 patients with open tibia fracture	HBOT, 2.4 to 2.8 ATA, 80 to 100 min duration, both multiplace and monoplace chambers, 12 sessions over approximately nine days	Fracture and wound management surgery (debridement, excision, fasciotomy)	HBOT (60) vs. control (60)	necrosis, acute infection rate, LOS
Raj et al. [24]	2023	RCT, single center	India	2019 to 2022	60 patients with open fracture associated with severe soft tissue injury	HBOT, 2.4 ATA, 90 min, monoplace chamber, 12 session over nine days	Wound debridement, bone fixation, repair of nerve and tendon,	HBOT (30) vs. control (30)	wound size, depth, granulation, severity, infection, LOS
Chang et al. [17]	2024	observational, single center	Taiwan	2018 to 2021	72 patients with hand crushing injury	HBOT, inhaled 100% oxygen, 2.0 to 2.5 ATA, multiplace chamber, 90 to 120 min duration, 10 to 20 times session	Wound debridement, NPWT, revascularization, neurorrhaphy, tenorrhaphy, bone fixation, and possible wound repair	Overall HBOT (36) vs. control (36) ^1^	infection, wound healing, length of hospital stay, time to wound healing
Marra et al. [25]	2024	observational, single center	Italy	2010 to 2021	57 patients with extensive trauma of the lower limbs	HBOT, 2.5 ATA, 24.8 sessions, 80 to 90 min duration	Wound debridement, NPWT, split thickness skin graft, and soft tissue reconstruction	HBOT (36) vs. control (21)	infection, graft loss, time to complete healing

HBOT, hyperbaric oxygen therapy; ATA, absolute atmosphere, RCT, randomized control trial; NATO, North Atlantic Treaty Organization; NPWT, negative pressure wound therapy; LOS, length of hospital stay (days); ^1^ Chang et al. reported time-to-healing and wound-infection outcomes separately according to wound area. The >50 cm^2^ subgroup comprised 20 HBOT-treated patients and 8 controls and was used in the primary meta-analysis because it was considered more representative of severe traumatic limb injury. The ≤50 cm^2^ subgroup comprised 16 HBOT-treated patients and 28 controls and was evaluated in sensitivity analyses.

**Table 2:** Excluded studies.

Author	Year	Study Design	Location	Study Period	Patients	Intervention	Comparison	Outcome	Reason for Exclusion
Monies-Chass et al. [26]	1977	Observational case series, single-center	Israel	During the last 5 years (prior to publication)	7 patients with severe vascular trauma and acute ischemia of the lower limbs despite technically successful vascular repairs.	Adjuvant HBOT at 2.8 atm.	None	Prevented the development of gangrene in all cases; led to the disappearance of ischemia.	No eligible comparator; case series
Butler et al. [28]	1987	RCT	United Kingdom	Not described	39 patients undergoing below-knee amputation	Adjuvant oxygen therapy (28% normobaric oxygen for 48 h)	Untreated control group	Prevented post-operative fall in transcutaneous pO_2_, improved stump healing, and reduced amputation failure rates.	Ineligible intervention and population: normobaric oxygen after amputation
Shupak et al. [27]	1987	Observational case series, single-center	Israel	January 1982–November 1986	13 patients with acute posttraumatic ischemia of the lower extremities unresponsive to surgical measures.	Adjuvant HBOT at 2.4 ATA.	None	Complete limb salvage was achieved in 61.5% (8/13) of cases, lowering of ischemia level distally in 30.7% (4/13), and no improvement in 7.7% (1/13).	No eligible comparator; case series
Mathieu et al. [29]	1990	Retrospective, single center	France	36-month period	23 patients with post-traumatic limb ischemia	HBOT (2.5 ATA)	Normal air vs. Normobaric pure oxygen vs. HBOT	Transcutaneous oxygen pressure ratio during HBOT successfully predicted the final outcome (amputation vs. recovery).	Ineligible outcome; prognostic transcutaneous oxygen measurements rather than comparative clinical outcomes
Huang et al. [30]	2007	Prospective observational, single center	Taiwan	January 2002–June 2004	16 patients with complex open elbow injuries	HBOT + immediate internal fixation, debridement, and soft-tissue coverage	None	75% achieved satisfactory functional results (Mayo score) and no deep infections occurred.	No eligible comparator
D’Agostino Dias et al. [31]	2008	Retrospective, single center	Brazil	1992–2002	1506 cases (1014 acute injuries, 492 chronic injuries)	Adjuvant HBOT	Acute vs. chronic injuries/Different severity levels	Evaluated mortality rates and determined the number of sessions required for clinical resolution based on injury type and severity.	No eligible comparator and insufficient comparative data
Chiang et al. [32]	2017	Retrospective, single center	Taiwan	January 2006–December 2014	45 patients with mutilated hand injuries	Adjunctive HBOT after reconstruction or revascularization	None	81% replanted finger survival rate, 100% palm survival rate; HBOT was effective in preserved partially viable tissue and restoring hand function.	No eligible comparator
De Francesco et al. [33]	2020	Retrospective, single center	Italy	2006–2018	179 patients with contaminated multi-tissue injuries (mangled upper limbs)	NPWT + HBOT	NPWT alone	NPWT + HBOT combination resulted in significantly higher wound closure rates (97%) and lower postoperative pain compared to NPWT alone.	No eligible comparator
Jirangkul et al. [35]	2021	Prospective observational, single center	Thailand	January 2008–January 2018	18 patients with severe mangled extremities (MESS ≥ 7)	Adjuvant HBOT following current standard surgical treatment	None	Successful limb salvage in most cases (only 1 amputation), minimal soft tissue infection, and favorable clinical findings.	No eligible comparator
Ince et al. [36]	2022	RCT	Turkey	2015–2019	74 patients with upper extremity nerve injuries (ulnar and median)	HBOT + standard epineural nerve repair	HBOT + surgery vs. Epineural nerve repair alone	Demonstrated faster impulse transmission and more rapid motor recovery/muscle strength scores in HBOT patients.	Ineligible outcome: peripheral nerve recovery
Oley et al. [34]	2021	RCT	Indonesia	5-year period	15 patients with crush injury	HBOT + initial treatment (debridement and limb-salvage surgeries)	HBOT vs. conventional treatment	Significantly increased VEGF serum levels and VEGF mRNA expression in HBOT, which accelerates wound healing.	Ineligible outcome: VEGF mRNA expression
Ergani et al. [37]	2023	Retrospective, single center	Türkiye	6–15 February 2023	120 earthquake victims (75 underwent fasciotomy)	HBOT following fasciotomy	None (Observational)	Proper triage and interventions, including HBOT, reduced the amputation rate and yielded clinically satisfactory results.	No eligible comparator
Casimiro et al. [39]	2024	Retrospective, multicenter (two hopsitals)	Portugal	April 2014–May 2015	19 adult patients with upper limb crush injuries (Gustilo III)	Adjunctive HBOT	None	Evaluated trauma-related acute complications (tissue necrosis, local infection) and late complications (pseudarthrosis, late deep infection).	No eligible comparator
Demir & Öztürk et al. [38]	2024	Retrospective, single center	Türkiye	Not described	35 patients with severe earthquake-related injuries (MESS 7-14)	HBOT	None	Achieved sensory recovery in 54.3% and functional recovery in 51.4% of patients; evaluated minor and major amputation rates.	No eligible comparator

HBOT, hyperbaric oxygen therapy; ATA, absolute atmosphere; atm, standard atmosphere; RCT, randomized control trial; NPWT, negative pressure wound therapy; MESS, mangled extremity severity score.

**Table 3:** Risk of bias assessment of randomized studies—RoB 2.0.

Author	Year	Bias Arising from the Randomization Process	Bias Due to Deviations from Intended Interventions	Bias Due to Missing Outcome Data	Bias in Measurement of the Outcome	Bias in Selection of the Reported Result	Overall Bias
Bouachour et al. [20]	1996	HIGH	LOW	LOW	LOW	SC	HIGH
Millar et al. [23]	2022	LOW	LOW	LOW	LOW	LOW	LOW
Raj et al. [24]	2023	SC	LOW	LOW	LOW	SC	HIGH

RoB 2, Revised Cochrane risk-of-bias tool for randomized trials; SC, some concerns.

**Table 4:** The risk of bias assessment of included papers using ROBINS-I tool for non-randomized studies.

Study		Pre-Intervention		At Intervention		Post-Intervention		Overall Risk of Bias	
Author	Year	Bias Due to Confounding	Bias in Selection of Participants into the Study	Bias in Classification of Interventions	Bias Due to Deviations from Intended Interventions	Bias Due to Missing Data	Bias in Measurement of Outcomes	Bias in Selection of the Reported Result	Low/Moderate/Serious/Critical/No Information
Roje et al. [21]	2008	SERIOUS	LOW	LOW	NI	LOW	LOW	MODERATE	SERIOUS
Yamada et al. [22]	2014	SERIOUS	MODERATE	LOW	NI	LOW	LOW	MODERATE	SERIOUS
Chang et al. [17]	2024	SERIOUS	LOW	LOW	NI	LOW	LOW	MODERATE	SERIOUS
Marra et al. [25]	2024	SERIOUS	LOW	LOW	NI	LOW	LOW	MODERATE	SERIOUS

ROBINS-I, Risk Of Bias In Non-randomized Studies of Interventions; NI, no information.

**Table 5:** GRADE Summary of findings for adjunctive hyperbaric oxygen therapy in severe traumatic limb injuries.

Outcome	No. of Studies	Study Design	Risk of Bias	Inconsistency	Indirectness	Imprecision	Other Considerations	HBOT + Standard Care	Control/Placebo + Standard Care	Relative Effect (95% CI)	Absolute Effect (95% CI)	Certainty	Importance
**Complete wound healing**	1	Randomised trials	Serious ^a^	Not serious	Not serious	Very serious ^b^	None	17/18 (94.4%)	10/18 (55.6%)	OR 13.60 (1.48 to 125.32) ^c^	389 more per 1000 (94 more to 438 more)	⊕◯◯◯ Very low	Critical
**Time to wound healing—RCT**	1	Randomised trials	Serious ^d^	Not serious	Not serious	Very serious ^e^	None ^f^	18	18	—	MD 5.60 days lower (19.00 lower to 7.80 higher)	⊕◯◯◯ Very low	Critical
**Time to wound healing—observational**	2	Non-randomised studies	Serious ^g^	Not serious	Not serious	Serious ^h^	None ^f^	56	29	—	MD 0.69 days lower (6.40 lower to 5.03 higher)	⊕◯◯◯ Very low	Critical
**Length of hospital stay—RCTs**	3	Randomised trials	Serious ^i^	Very serious ^j^	Not serious	Serious ^k^	None ^f^	108	108	—	MD 6.93 days lower (20.97 lower to 7.12 higher)	⊕◯◯◯ Very low	Critical
**Length of hospital stay—observational**	2	Non-randomised studies	Serious ^l^	Not serious	Not serious	Serious ^m^	None ^f^	36	21	—	MD 3.85 days higher (5.98 lower to 13.68 higher)	⊕◯◯◯ Very low	Critical
**Wound infection—RCTs**	3	Randomised trials	Serious ^n^	Not serious	Not serious	Serious ^o^	None ^f^	16/106 (15.1%)	29/107 (27.1%)	OR 0.46 (0.23 to 0.93)	125 fewer per 1000 (192 fewer to 14 fewer)	⊕⊕◯◯ Low	Critical
**Wound infection—observational**	4	Non-randomised studies	Serious ^p^	Not serious	Not serious	Not serious	None ^f^	49/171 (28.7%)	213/331 (64.4%)	OR 0.27 (0.18 to 0.41)	316 fewer per 1000 (398 fewer to 218 fewer)	⊕◯◯◯ Very low	Critical
**Wound necrosis—RCTs**	2	Randomised trials	Serious ^q^	Serious ^r^	Not serious	Very serious ^s^	None ^f^	18/76 (23.7%)	41/77 (53.2%)	OR 0.17 (0.02 to 1.26)	370 fewer per 1000 (510 fewer to 57 more)	⊕◯◯◯ Very low	Critical
**Wound necrosis—observational**	2	Non-randomised studies	Serious ^t^	Not serious	Not serious	Not serious	None ^f^	19/135 (14.1%)	152/310 (49.0%)	OR 0.20 (0.11 to 0.38)	329 fewer per 1000 (395 fewer to 223 fewer)	⊕◯◯◯ Very low	Critical

Notes: ^a^ Downgraded one level for serious risk of bias because the only study contributing to this outcome was judged to have a high overall risk of bias, primarily due to concerns regarding the randomization process and selection of the reported result. ^b^ Downgraded two levels for very serious imprecision because the estimate was derived from a single small trial with only 36 participants, did not meet the optimal information size, and had an extremely wide confidence interval, indicating substantial uncertainty regarding the magnitude of the effect. ^c^ The odds ratio and 95% confidence interval were calculated from the event data reported in the original study. ^d^ Downgraded one level for serious risk of bias because the single contributing randomized trial was judged to have a high overall risk of bias, primarily owing to concerns regarding the randomization process and selection of the reported result. ^e^ Downgraded two levels for very serious imprecision because the estimate was based on a single small trial with 36 participants, did not meet the optimal information size, and the confidence interval included both a clinically important reduction and a possible increase in time to wound healing. ^f^ Publication bias could not be formally assessed because of the small number of studies; therefore, no downgrade was applied. ^g^ Downgraded one level for serious risk of bias because both contributing non-randomized studies had serious concerns regarding confounding and baseline imbalances. ^h^ Downgraded one level for serious imprecision because the confidence interval crossed the line of no effect and included both a potentially clinically important reduction and a possible increase in time to wound healing. ^i^ Downgraded one level for serious risk of bias because two of the three contributing randomized trials were judged to have a high overall risk of bias. ^j^ Downgraded two levels for very serious inconsistency because there was substantial unexplained heterogeneity among the randomized trials (I^2^ = 96%), with markedly divergent effect estimates. ^k^ Downgraded one level for serious imprecision because the confidence interval crossed the line of no effect and included both a clinically important reduction and little or no difference in length of hospital stay. ^l^ Downgraded one level for serious risk of bias because both contributing non-randomized studies had serious concerns regarding confounding and baseline differences between the treatment groups. ^m^ Downgraded one level for serious imprecision because the confidence interval crossed the line of no effect and included both a reduction and an increase in length of hospital stay. ^n^ Downgraded one level for serious risk of bias because two of the three contributing randomized trials were judged to have a high overall risk of bias. ^o^ Downgraded one level for serious imprecision because the estimate was based on a limited number of participants and events, and the confidence interval included effects ranging from a substantial to a potentially small reduction in wound infection. ^p^ Downgraded one level for serious risk of bias because all four contributing non-randomized studies had serious concerns regarding confounding and baseline differences between the treatment groups. ^q^ Downgraded one level for serious risk of bias because one of the two contributing randomized trials had a high overall risk of bias. ^r^ Downgraded one level for serious inconsistency because substantial heterogeneity was observed between the randomized trials (I^2^ = 67%), although both effect estimates favored HBOT. ^s^ Downgraded two levels for very serious imprecision because the estimate was based on a small number of participants and events, and the very wide confidence interval included both a substantial reduction in wound necrosis and a possible increase. ^t^ Downgraded one level for serious risk of bias because both contributing non-randomized studies had serious concerns regarding confounding and baseline differences between groups. Abbreviations: CI, confidence interval; HBOT, hyperbaric oxygen therapy; MD, mean difference; OR, odds ratio; RCT, randomized controlled trial.

## Data Availability

All data analysed during this study are included in this published article and its Supplementary Information files.

## Supplementary Materials

The following supporting information can be downloaded at: https://www.mdpi.com/article/10.3390/medicina62081589/s1. Supplementary Table S1: PRISMA 2020 Checklist; Supplementary Table S2: Full search strategies for PubMed, Embase, and Cochrane Library; Supplementary Figures S1–S5: Leave-one-out sensitivity analysis for clinical outcomes.
