# Supplementary material for: Adjunctive Hyperbaric Oxygen Therapy for Severe Traumatic Limb Injuries: A Systematic Review and Meta-Analysis
Source: Medicina (Kaunas). 2026 Aug 18;62(8):1589. doi: 10.3390/medicina62081589 (PMC13515450; doi:10.3390/medicina62081589)

Supplementary Table S1. PRISMA_2020_checklist

	Item #	Checklist item	Location where item is reported
**TITLE**			
Title	1	Identify the report as a systematic review.	1
**ABSTRACT**			
Abstract	2	See the PRISMA 2020 for Abstracts checklist.	1
**INTRODUCTION**			
Rationale	3	Describe the rationale for the review in the context of existing knowledge.	1-2
Objectives	4	Provide an explicit statement of the objective(s) or question(s) the review addresses.	1-2
**METHODS**			
Eligibility criteria	5	Specify the inclusion and exclusion criteria for the review and how studies were grouped for the syntheses.	2-3
Information sources	6	Specify all databases, registers, websites, organisations, reference lists and other sources searched or consulted to identify studies. Specify the date when each source was last searched or consulted.	2-3
Search strategy	7	Present the full search strategies for all databases, registers and websites, including any filters and limits used.	2
Selection process	8	Specify the methods used to decide whether a study met the inclusion criteria of the review, including how many reviewers screened each record and each report retrieved, whether they worked independently, and if applicable, details of automation tools used in the process.	2
Data collection process	9	Specify the methods used to collect data from reports, including how many reviewers collected data from each report, whether they worked independently, any processes for obtaining or confirming data from study investigators, and if applicable, details of automation tools used in the process.	2-3
Data items	10a	List and define all outcomes for which data were sought. Specify whether all results that were compatible with each outcome domain in each study were sought (e.g. for all measures, time points, analyses), and if not, the methods used to decide which results to collect.	2-3
	10b	List and define all other variables for which data were sought (e.g. participant and intervention characteristics, funding sources). Describe any assumptions made about any missing or unclear information.	2-3
Study risk of bias assessment	11	Specify the methods used to assess risk of bias in the included studies, including details of the tool(s) used, how many reviewers assessed each study and whether they worked independently, and if applicable, details of automation tools used in the process.	3
Effect measures	12	Specify for each outcome the effect measure(s) (e.g. risk ratio, mean difference) used in the synthesis or presentation of results.	2-3
Synthesis methods	13a	Describe the processes used to decide which studies were eligible for each synthesis (e.g. tabulating the study intervention characteristics and comparing against the planned groups for each synthesis (item #5)).	2-3
	13b	Describe any methods required to prepare the data for presentation or synthesis, such as handling of missing summary statistics, or data conversions.	Not applicable
	13c	Describe any methods used to tabulate or visually display results of individual studies and syntheses.	2-3
	13d	Describe any methods used to synthesize results and provide a rationale for the choice(s). If meta-analysis was performed, describe the	2-3

	Item #	Checklist item	Location where item is reported
		model(s), method(s) to identify the presence and extent of statistical heterogeneity, and software package(s) used.	
	13e	Describe any methods used to explore possible causes of heterogeneity among study results (e.g. subgroup analysis, meta-regression).	2-3
	13f	Describe any sensitivity analyses conducted to assess robustness of the synthesized results.	2-3
Reporting bias assessment	14	Describe any methods used to assess risk of bias due to missing results in a synthesis (arising from reporting biases).	3
Certainty assessment	15	Describe any methods used to assess certainty (or confidence) in the body of evidence for an outcome.	Not applicable
**RESULTS**			
Study selection	16a	Describe the results of the search and selection process, from the number of records identified in the search to the number of studies included in the review, ideally using a flow diagram.	3-4
	16b	Cite studies that might appear to meet the inclusion criteria, but which were excluded, and explain why they were excluded.	3-4
Study characteristics	17	Cite each included study and present its characteristics.	6-12
Risk of bias in studies	18	Present assessments of risk of bias for each included study.	8
Results of individual studies	19	For all outcomes, present, for each study: (a) summary statistics for each group (where appropriate) and (b) an effect estimate and its precision (e.g. confidence/credible interval), ideally using structured tables or plots.	13-16
Results of syntheses	20a	For each synthesis, briefly summarise the characteristics and risk of bias among contributing studies.	15-17
	20b	Present results of all statistical syntheses conducted. If meta-analysis was done, present for each the summary estimate and its precision (e.g. confidence/credible interval) and measures of statistical heterogeneity. If comparing groups, describe the direction of the effect.	15-17
	20c	Present results of all investigations of possible causes of heterogeneity among study results.	15-17
	20d	Present results of all sensitivity analyses conducted to assess the robustness of the synthesized results.	15-17
Reporting biases	21	Present assessments of risk of bias due to missing results (arising from reporting biases) for each synthesis assessed.	Not applicable
Certainty of evidence	22	Present assessments of certainty (or confidence) in the body of evidence for each outcome assessed.	Not applicable
**DISCUSSION**			
Discussion	23a	Provide a general interpretation of the results in the context of other evidence.	17-18
	23b	Discuss any limitations of the evidence included in the review.	19
	23c	Discuss any limitations of the review processes used.	19
	23d	Discuss implications of the results for practice, policy, and future research.	18-19

	Item #	Checklist item	Location where item is reported
**OTHER INFORMATION**			
Registration and protocol	24a	Provide registration information for the review, including register name and registration number, or state that the review was not registered.	2
	24b	Indicate where the review protocol can be accessed, or state that a protocol was not prepared.	2
	24c	Describe and explain any amendments to information provided at registration or in the protocol.	2
Support	25	Describe sources of financial or non-financial support for the review, and the role of the funders or sponsors in the review.	18
Competing interests	26	Declare any competing interests of review authors.	19
Availability of data, code and other materials	27	Report which of the following are publicly available and where they can be found: template data collection forms; data extracted from included studies; data used for all analyses; analytic code; any other materials used in the review.	19

Supplementary Table S2. Search strategy

Database	MEDLINE PubMed: title, abstract
Searching date	up to May 7, 2026
Query	
#1	Hyperbaric*
#2	oxygen inhalation therapy
#3	hyperbaric oxygen therapy
#4	HBO
#5	HBOT
#6	#1 OR #2 OR #3 OR #4 OR #5
#7	trauma*
#8	injur*
#9	crush*
#10	wound*
#11	fract*
#12	mangl*
#13	soft tissue injur*
#14	#7 OR #8 OR #9 OR #10 OR #11 OR #12 OR #13
#15	extremit*
#16	limb
#17	muscle*
#18	Musculo*
#19	skeletal*
#20	hand injury
#21	lower extremity
#22	hand injuries
#23	#15 OR #16 OR #17 OR #18 OR #19 OR #20 OR #21 OR #22
#24	#6 AND #14 AND #23

Database	Embase: title, abstract, keywords
Searching date	up to May 7, 2026
Query	
#1	Hyperbaric*
#2	oxygen inhalation therapy
#3	hyperbaric oxygen therapy
#4	HBO
#5	HBOT
#6	#1 OR #2 OR #3 OR #4 OR #5
#7	trauma*
#8	injur*
#9	crush*
#10	wound*
#11	fract*

#12	mangl*
#13	soft tissue injur*
#14	#7 OR #8 OR #9 OR #10 OR #11 OR #12 OR #13
#15	extremit*
#16	limb
#17	muscle*
#18	Musculo*
#19	skeletal*
#20	hand injury
#21	lower extremity
#22	hand injuries
#23	#15 OR #16 OR #17 OR #18 OR #19 OR #20 OR #21 OR #22
#24	#6 AND #14 AND #23

Database	Cochrane: title, abstract, keywords
Searching date	up to May 7, 2026
Query	
#1	Hyperbaric*
#2	oxygen inhalation therapy
#3	hyperbaric oxygen therapy
#4	HBO
#5	HBOT
#6	#1 OR #2 OR #3 OR #4 OR #5
#7	trauma*
#8	injur*
#9	crush*
#10	wound*
#11	fract*
#12	mangl*
#13	soft tissue injur*
#14	#7 OR #8 OR #9 OR #10 OR #11 OR #12 OR #13
#15	extremit*
#16	limb
#17	muscle*
#18	Musculo*
#19	skeletal*
#20	hand injury
#21	lower extremity
#22	hand injuries
#23	#15 OR #16 OR #17 OR #18 OR #19 OR #20 OR #21 OR #22
#24	#6 AND #14 AND #23

Supplementary Figure S1. Sensitivity analysis according to time to wound healing (days). Sensitivity analysis by omitting each study. (A) Omitting study by Bouachour et al., (B) Omitting study by Chang et al., and (C) Omitting study by Marra et al..

(A)

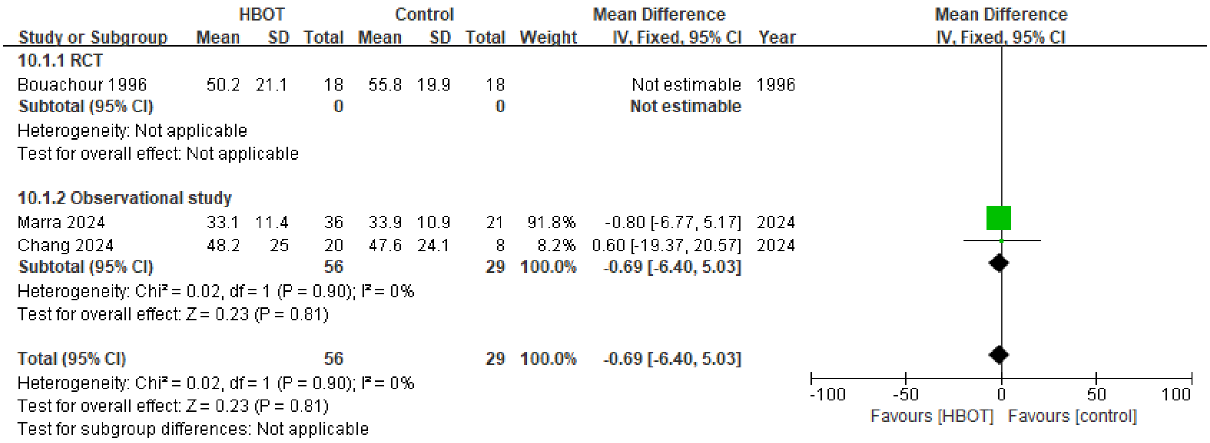

(B)

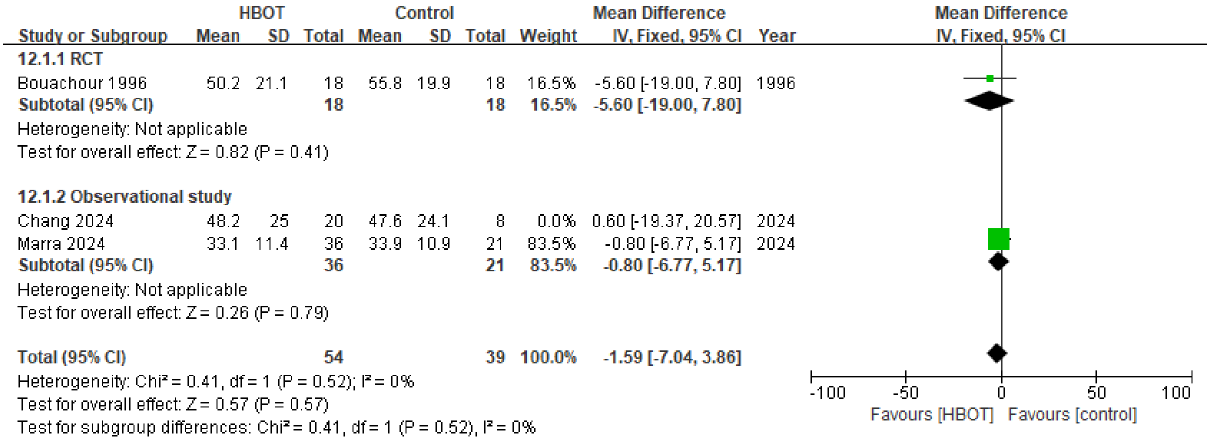

(C)

Study or Subgroup	HBOT Mean	HBOT SD	HBOT Total	Control Mean	Control SD	Control Total	Weight	Mean Difference IV, Fixed, 95% CI	Year
**11.1.1 RCT**									
Bouachour 1996	50.2	21.1	18	55.8	19.9	18	69.0%	-5.60 [-19.00, 7.80]	1996
**Subtotal (95% CI)**			**18**			**18**	**69.0%**	**-5.60 [-19.00, 7.80]**	
Heterogeneity: Not applicable									
Test for overall effect: Z = 0.82 (P = 0.41)									
**11.1.2 Observational study**									
Chang 2024	48.2	25	20	47.6	24.1	8	31.0%	0.60 [-19.37, 20.57]	2024
Marra 2024	33.1	11.4	36	33.9	10.9	21	0.0%	-0.80 [-6.77, 5.17]	2024
**Subtotal (95% CI)**			**20**			**8**	**31.0%**	**0.60 [-19.37, 20.57]**	
Heterogeneity: Not applicable									
Test for overall effect: Z = 0.06 (P = 0.95)									
**Total (95% CI)**			**38**			**26**	**100.0%**	**-3.68 [-14.80, 7.45]**	
Heterogeneity: Chi² = 0.26, df = 1 (P = 0.61); I² = 0%									
Test for overall effect: Z = 0.65 (P = 0.52)									
Test for subgroup differences: Chi² = 0.26, df = 1 (P = 0.61), I² = 0%									

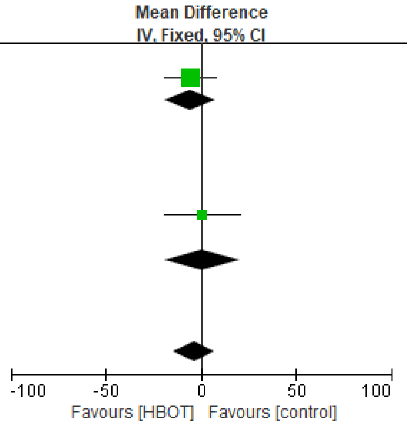

Supplementary Figure S2. Sensitivity analysis according to length of hospital stays (days). Sensitivity analysis by omitting each study. (A) Omitting study by Bouachour et al. (B) Omitting study by Millar et al., (C) Omitting study by Raj et al., (D) Omitting study by Yamada et al., and (E) Omitting study by Chang et al..

(A)

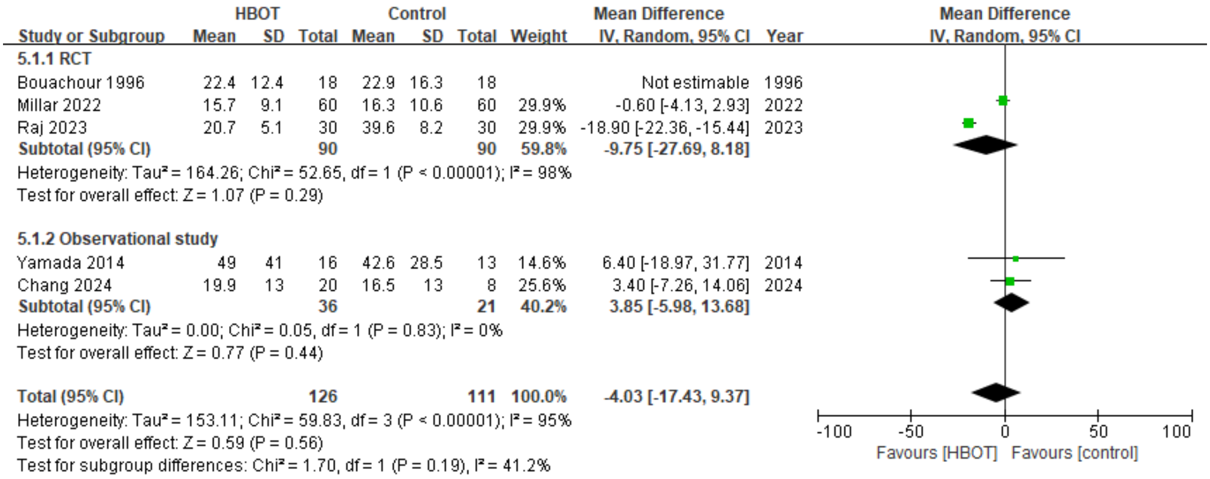

(B)

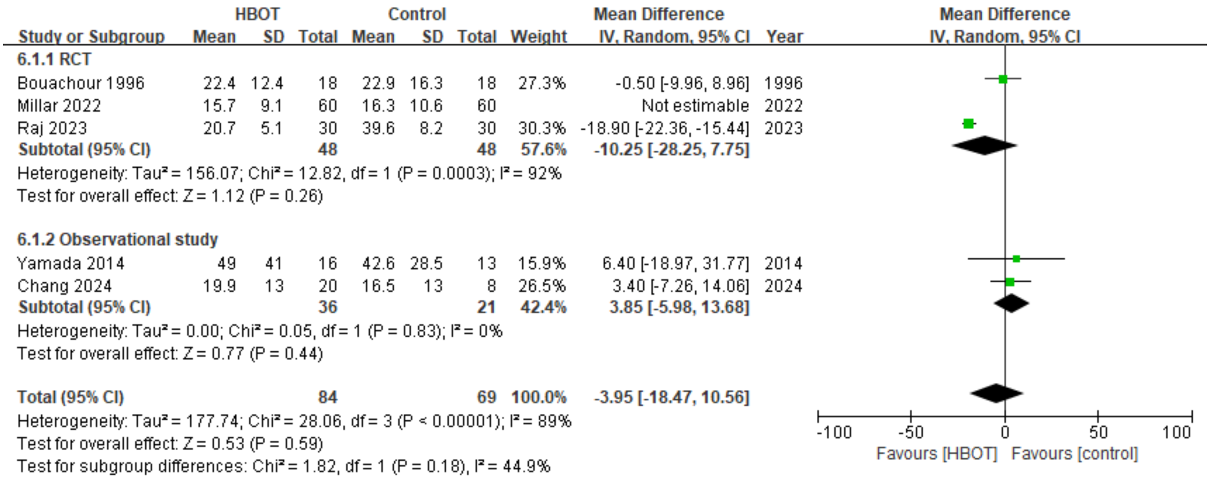

(C)

Study or Subgroup	HBOT Mean	HBOT SD	HBOT Total	Control Mean	Control SD	Control Total	Weight	Mean Difference IV, Fixed, 95% CI	Year
**7.1.1 RCT**									
Bouachour 1996	22.4	12.4	18	22.9	16.3	18	11.0%	-0.50 [-9.96, 8.96]	1996
Millar 2022	15.7	9.1	60	16.3	10.6	60	78.8%	-0.60 [-4.13, 2.93]	2022
Raj 2023	20.7	5.1	30	39.6	8.2	30		Not estimable	2023
**Subtotal (95% CI)**			**78**			**78**	**89.8%**	**-0.59 [-3.90, 2.72]**	
Heterogeneity: Chi^2 = 0.00, df = 1 (P = 0.98); I^2 = 0%									
Test for overall effect: Z = 0.35 (P = 0.73)									
**7.1.2 Observational study**									
Yamada 2014	49	41	16	42.6	28.5	13	1.5%	6.40 [-18.97, 31.77]	2014
Chang 2024	19.9	13	20	16.5	13	8	8.7%	3.40 [-7.26, 14.06]	2024
**Subtotal (95% CI)**			**36**			**21**	**10.2%**	**3.85 [-5.98, 13.68]**	
Heterogeneity: Chi^2 = 0.05, df = 1 (P = 0.83); I^2 = 0%									
Test for overall effect: Z = 0.77 (P = 0.44)									
**Total (95% CI)**			**114**			**99**	**100.0%**	**-0.14 [-3.27, 3.00]**	
Heterogeneity: Chi^2 = 0.75, df = 3 (P = 0.86); I^2 = 0%									
Test for overall effect: Z = 0.08 (P = 0.93)									
Test for subgroup differences: Chi^2 = 0.70, df = 1 (P = 0.40), I^2 = 0%									

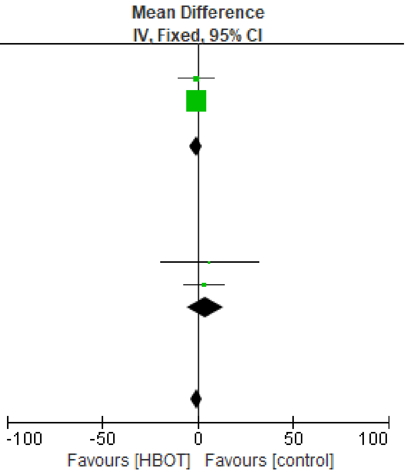

(D)

Study or Subgroup	HBOT Mean	HBOT SD	HBOT Total	Control Mean	Control SD	Control Total	Weight	Mean Difference IV, Random, 95% CI	Year
**8.1.1 RCT**									
Bouachour 1996	22.4	12.4	18	22.9	16.3	18	23.6%	-0.50 [-9.96, 8.96]	1996
Millar 2022	15.7	9.1	60	16.3	10.6	60	26.9%	-0.60 [-4.13, 2.93]	2022
Raj 2023	20.7	5.1	30	39.6	8.2	30	26.9%	-18.90 [-22.36, -15.44]	2023
**Subtotal (95% CI)**			**108**			**108**	**77.3%**	**-6.93 [-20.97, 7.12]**	
Heterogeneity: Tau^2 = 144.76; Chi^2 = 56.24, df = 2 (P < 0.00001); I^2 = 96%									
Test for overall effect: Z = 0.97 (P = 0.33)									
**8.1.2 Observational study**									
Yamada 2014	49	41	16	42.6	28.5	13		Not estimable	2014
Chang 2024	19.9	13	20	16.5	13	8	22.7%	3.40 [-7.26, 14.06]	2024
**Subtotal (95% CI)**			**20**			**8**	**22.7%**	**3.40 [-7.26, 14.06]**	
Heterogeneity: Not applicable									
Test for overall effect: Z = 0.63 (P = 0.53)									
**Total (95% CI)**			**128**			**116**	**100.0%**	**-4.59 [-16.70, 7.52]**	
Heterogeneity: Tau^2 = 138.86; Chi^2 = 61.48, df = 3 (P < 0.00001); I^2 = 95%									
Test for overall effect: Z = 0.74 (P = 0.46)									
Test for subgroup differences: Chi^2 = 1.32, df = 1 (P = 0.25), I^2 = 24.1%									

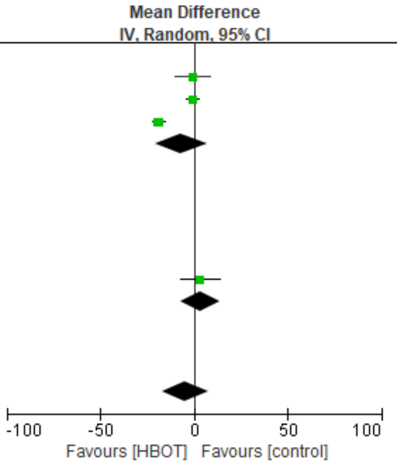

(E)

Study or Subgroup	HBOT Mean	HBOT SD	HBOT Total	Control Mean	Control SD	Control Total	Weight	Mean Difference IV, Random, 95% CI	Year
**9.1.1 RCT**									
Bouachour 1996	22.4	12.4	18	22.9	16.3	18	26.2%	-0.50 [-9.96, 8.96]	1996
Millar 2022	15.7	9.1	60	16.3	10.6	60	29.8%	-0.60 [-4.13, 2.93]	2022
Raj 2023	20.7	5.1	30	39.6	8.2	30	29.9%	-18.90 [-22.36, -15.44]	2023
**Subtotal (95% CI)**			**108**			**108**	**86.0%**	**-6.93 [-20.97, 7.12]**	
Heterogeneity: Tau^2 = 144.76; Chi^2 = 56.24, df = 2 (P < 0.00001); I^2 = 96%									
Test for overall effect: Z = 0.97 (P = 0.33)									
**9.1.2 Observational study**									
Yamada 2014	49	41	16	42.6	28.5	13	14.0%	6.40 [-18.97, 31.77]	2014
Chang 2024	19.9	13	20	16.5	13	8	0.0%	3.40 [-7.26, 14.06]	2024
**Subtotal (95% CI)**			**16**			**13**	**14.0%**	**6.40 [-18.97, 31.77]**	
Heterogeneity: Not applicable									
Test for overall effect: Z = 0.49 (P = 0.62)									
**Total (95% CI)**			**124**			**121**	**100.0%**	**-5.06 [-18.00, 7.88]**	
Heterogeneity: Tau^2 = 142.76; Chi^2 = 57.71, df = 3 (P < 0.00001); I^2 = 95%									
Test for overall effect: Z = 0.77 (P = 0.44)									
Test for subgroup differences: Chi^2 = 0.81, df = 1 (P = 0.37), I^2 = 0%									

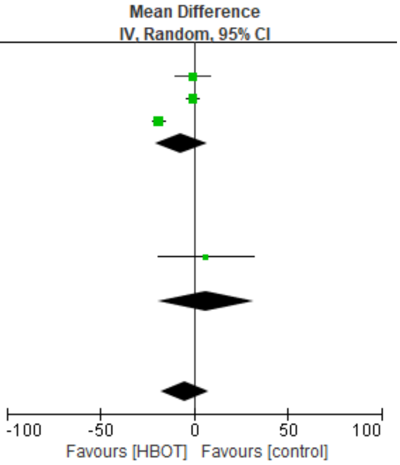

Supplementary Figure S3. Sensitivity analysis according to wound infection. Sensitivity analysis by omitting each study. (A) Omitting study by Bouachour et al., (B) Omitting study by Millar et al., (C) Omitting study by Raj et al., (D) Omitting study by Roje et al. (E) Omitting study by Yamada et al. (F) Omitting study by Marra et al., and (G) Omitting study by Chang et al.

(A)

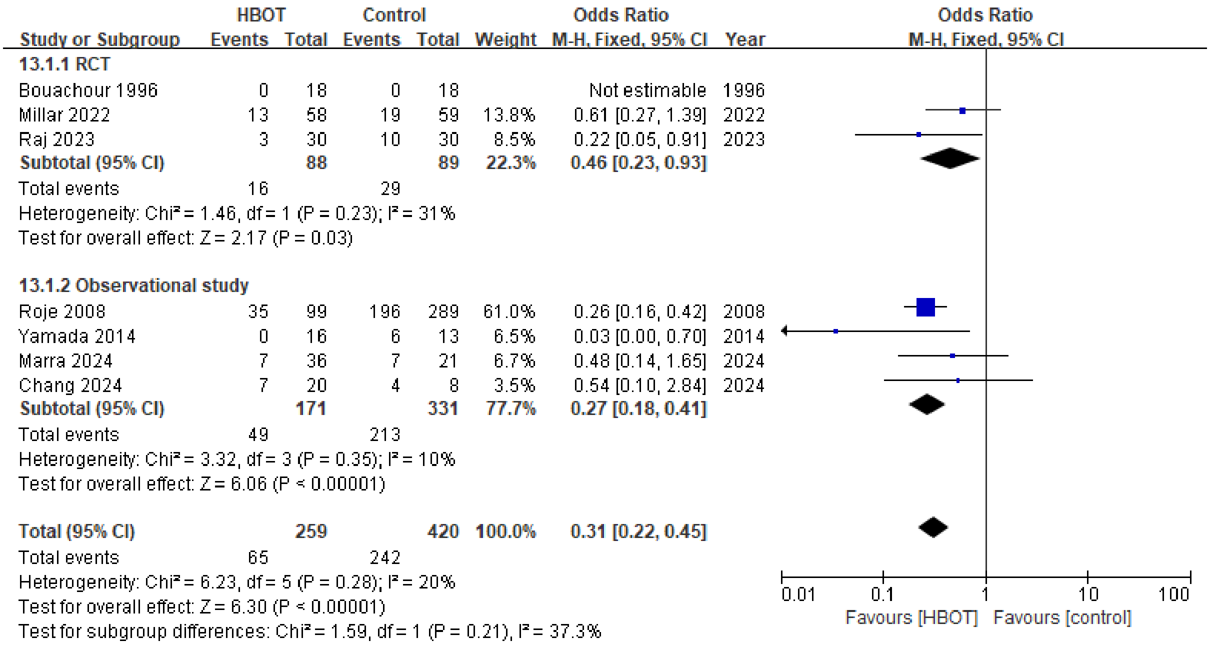

(B)

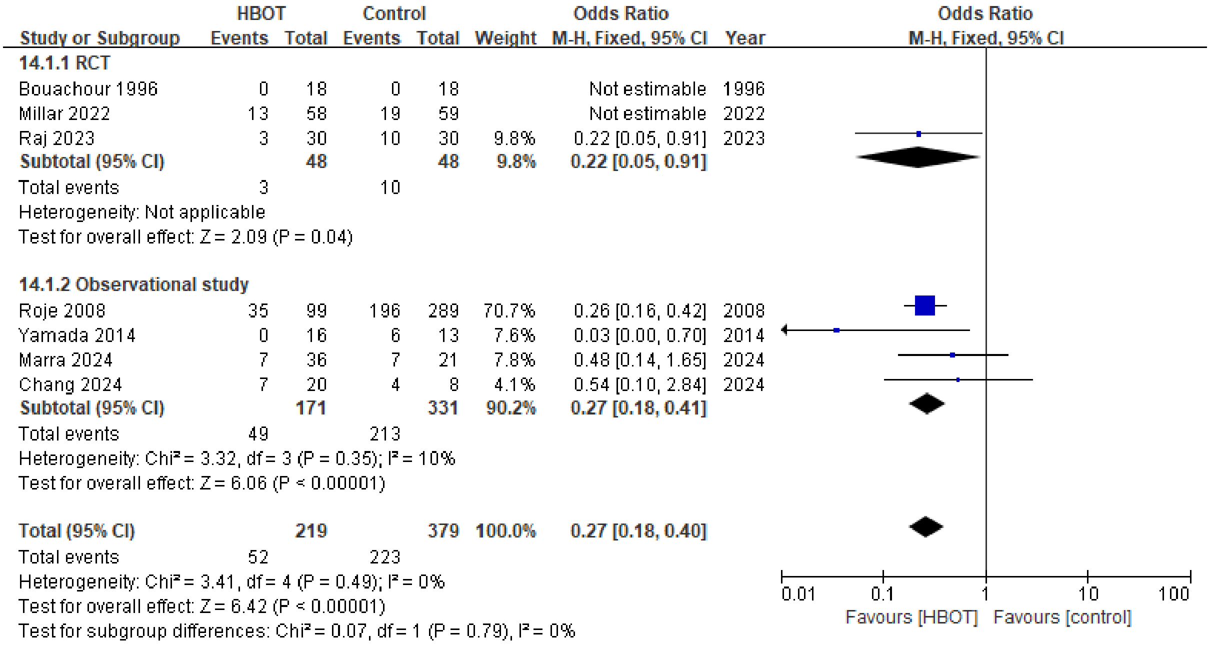
HBOT
Control
Odds Ratio
Odds Ratio
Study or Subgroup Events Total Events Total Weight M-H, Fixed, 95% CI Year
M-H, Fixed, 95% CI
14.1.1 RCT
Bouachour 1996 0 18 0 18 Not estimable 1996
Millar 2022 13 58 19 59 Not estimable 2022
Raj 2023 3 30 10 30 9.8% 0.22 [0.05, 0.91] 2023
Subtotal (95% CI) 48 48 9.8% 0.22 [0.05, 0.91]
Total events 3 10
Heterogeneity: Not applicable
Test for overall effect: Z = 2.09 (P = 0.04)
14.1.2 Observational study
Roje 2008 35 99 196 289 70.7% 0.26 [0.16, 0.42] 2008
Yamada 2014 0 16 6 13 7.6% 0.03 [0.00, 0.70] 2014
Marra 2024 7 36 7 21 7.8% 0.48 [0.14, 1.65] 2024
Chang 2024 7 20 4 8 4.1% 0.54 [0.10, 2.84] 2024
Subtotal (95% CI) 171 331 90.2% 0.27 [0.18, 0.41]
Total events 49 213
Heterogeneity: Chi² = 3.32, df = 3 (P = 0.35); I² = 10%
Test for overall effect: Z = 6.06 (P < 0.00001)
Total (95% CI) 219 379 100.0% 0.27 [0.18, 0.40]
Total events 52 223
Heterogeneity: Chi² = 3.41, df = 4 (P = 0.49); I² = 0%
Test for overall effect: Z = 6.42 (P < 0.00001)
Test for subgroup differences: Chi² = 0.07, df = 1 (P = 0.79), I² = 0%
0.01 0.1 1 10 100
Favours [HBOT] Favours [control]

(C)

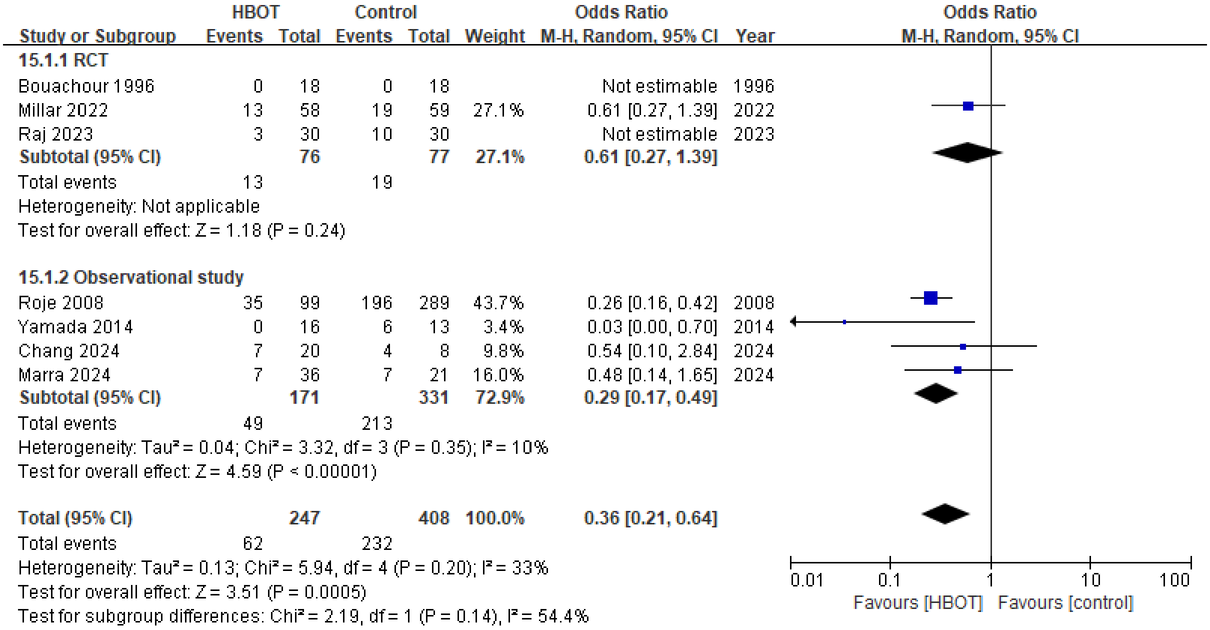
HBOT
Control
Odds Ratio
Odds Ratio
Study or Subgroup Events Total Events Total Weight M-H, Random, 95% CI Year
M-H, Random, 95% CI
15.1.1 RCT
Bouachour 1996 0 18 0 18 Not estimable 1996
Millar 2022 13 58 19 59 27.1% 0.61 [0.27, 1.39] 2022
Raj 2023 3 30 10 30 Not estimable 2023
Subtotal (95% CI) 76 77 27.1% 0.61 [0.27, 1.39]
Total events 13 19
Heterogeneity: Not applicable
Test for overall effect: Z = 1.18 (P = 0.24)
15.1.2 Observational study
Roje 2008 35 99 196 289 43.7% 0.26 [0.16, 0.42] 2008
Yamada 2014 0 16 6 13 3.4% 0.03 [0.00, 0.70] 2014
Chang 2024 7 20 4 8 9.8% 0.54 [0.10, 2.84] 2024
Marra 2024 7 36 7 21 16.0% 0.48 [0.14, 1.65] 2024
Subtotal (95% CI) 171 331 72.9% 0.29 [0.17, 0.49]
Total events 49 213
Heterogeneity: Tau² = 0.04; Chi² = 3.32, df = 3 (P = 0.35); I² = 10%
Test for overall effect: Z = 4.59 (P < 0.00001)
Total (95% CI) 247 408 100.0% 0.36 [0.21, 0.64]
Total events 62 232
Heterogeneity: Tau² = 0.13; Chi² = 5.94, df = 4 (P = 0.20); I² = 33%
Test for overall effect: Z = 3.51 (P = 0.0005)
Test for subgroup differences: Chi² = 2.19, df = 1 (P = 0.14), I² = 54.4%
0.01 0.1 1 10 100
Favours [HBOT] Favours [control]

(D)

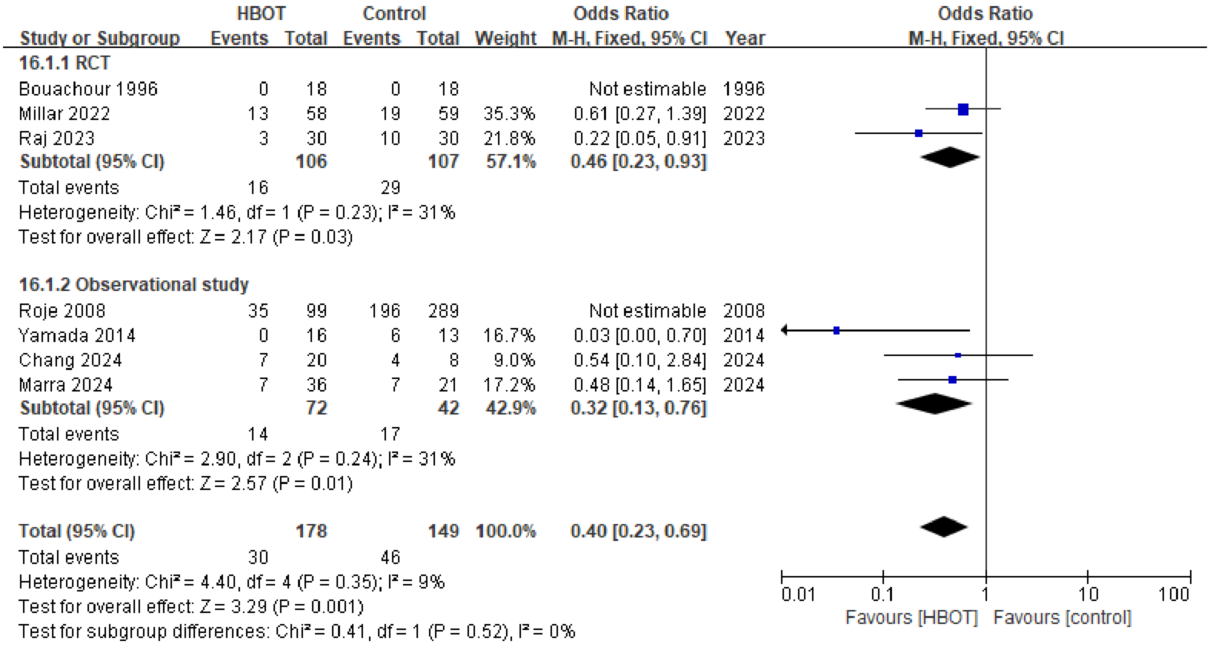
HBOT
Control
Odds Ratio
Odds Ratio
Study or Subgroup Events Total Events Total Weight M-H, Fixed, 95% CI Year
M-H, Fixed, 95% CI
16.1.1 RCT
Bouachour 1996 0 18 0 18 Not estimable 1996
Millar 2022 13 58 19 59 35.3% 0.61 [0.27, 1.39] 2022
Raj 2023 3 30 10 30 21.8% 0.22 [0.05, 0.91] 2023
Subtotal (95% CI) 106 107 57.1% 0.46 [0.23, 0.93]
Total events 16 29
Heterogeneity: Chi² = 1.46, df = 1 (P = 0.23); I² = 31%
Test for overall effect: Z = 2.17 (P = 0.03)
16.1.2 Observational study
Roje 2008 35 99 196 289 Not estimable 2008
Yamada 2014 0 16 6 13 16.7% 0.03 [0.00, 0.70] 2014
Chang 2024 7 20 4 8 9.0% 0.54 [0.10, 2.84] 2024
Marra 2024 7 36 7 21 17.2% 0.48 [0.14, 1.65] 2024
Subtotal (95% CI) 72 42 42.9% 0.32 [0.13, 0.76]
Total events 14 17
Heterogeneity: Chi² = 2.90, df = 2 (P = 0.24); I² = 31%
Test for overall effect: Z = 2.57 (P = 0.01)
Total (95% CI) 178 149 100.0% 0.40 [0.23, 0.69]
Total events 30 46
Heterogeneity: Chi² = 4.40, df = 4 (P = 0.35); I² = 9%
Test for overall effect: Z = 3.29 (P = 0.001)
Test for subgroup differences: Chi² = 0.41, df = 1 (P = 0.52), I² = 0%
0.01 0.1 1 10 100
Favours [HBOT] Favours [control]

(E)

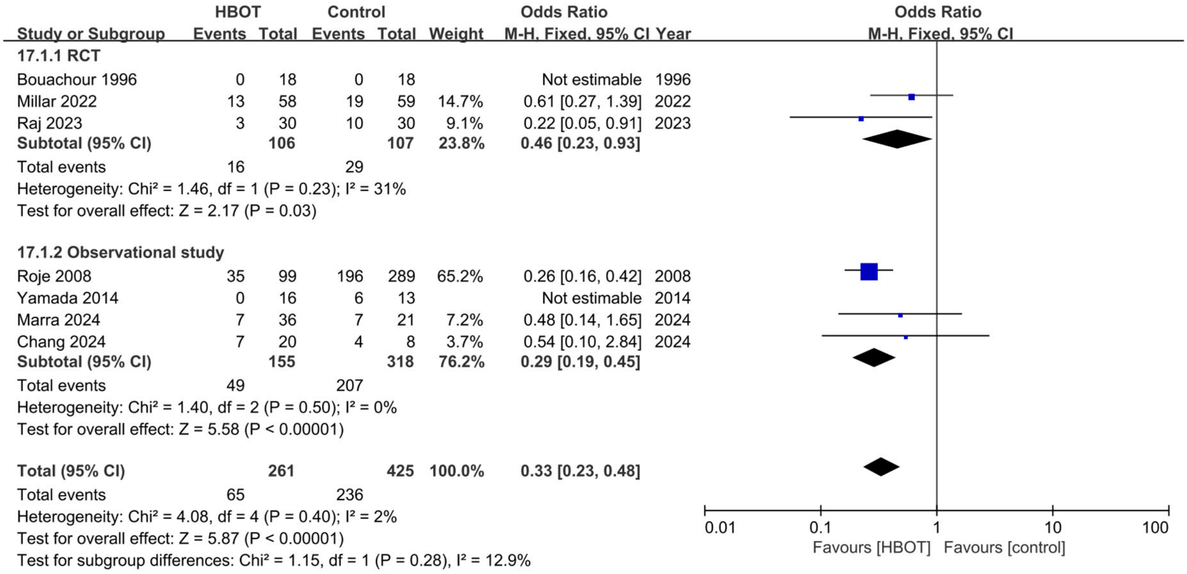
HBOT
Control
Odds Ratio
Odds Ratio
Study or Subgroup Events Total Events Total Weight M-H, Fixed, 95% CI Year
M-H, Fixed, 95% CI
17.1.1 RCT
Bouachour 1996 0 18 0 18 Not estimable 1996
Millar 2022 13 58 19 59 14.7% 0.61 [0.27, 1.39] 2022
Raj 2023 3 30 10 30 9.1% 0.22 [0.05, 0.91] 2023
Subtotal (95% CI) 106 107 23.8% 0.46 [0.23, 0.93]
Total events 16 29
Heterogeneity: Chi² = 1.46, df = 1 (P = 0.23); I² = 31%
Test for overall effect: Z = 2.17 (P = 0.03)
17.1.2 Observational study
Roje 2008 35 99 196 289 65.2% 0.26 [0.16, 0.42] 2008
Yamada 2014 0 16 6 13 Not estimable 2014
Marra 2024 7 36 7 21 7.2% 0.48 [0.14, 1.65] 2024
Chang 2024 7 20 4 8 3.7% 0.54 [0.10, 2.84] 2024
Subtotal (95% CI) 155 318 76.2% 0.29 [0.19, 0.45]
Total events 49 207
Heterogeneity: Chi² = 1.40, df = 2 (P = 0.50); I² = 0%
Test for overall effect: Z = 5.58 (P < 0.00001)
Total (95% CI) 261 425 100.0% 0.33 [0.23, 0.48]
Total events 65 236
Heterogeneity: Chi² = 4.08, df = 4 (P = 0.40); I² = 2%
Test for overall effect: Z = 5.87 (P < 0.00001)
Test for subgroup differences: Chi² = 1.15, df = 1 (P = 0.28), I² = 12.9%
0.01 0.1 1 10 100
Favours [HBOT] Favours [control]

(F)

Study or Subgroup	HBOT Events	HBOT Total	Control Events	Control Total	Weight	Odds Ratio M-H, Fixed, 95% CI	Year
**18.1.1 RCT**							
Bouachour 1996	0	18	0	18		Not estimable	1996
Millar 2022	13	58	19	59	14.8%	0.61 [0.27, 1.39]	2022
Raj 2023	3	30	10	30	9.1%	0.22 [0.05, 0.91]	2023
**Subtotal (95% CI)**		**106**		**107**	**23.9%**	**0.46 [0.23, 0.93]**	
Total events	16		29				
Heterogeneity: Chi² = 1.46, df = 1 (P = 0.23); I² = 31%							
Test for overall effect: Z = 2.17 (P = 0.03)							
**18.1.2 Observational study**							
Roje 2008	35	99	196	289	65.4%	0.26 [0.16, 0.42]	2008
Yamada 2014	0	16	6	13	7.0%	0.03 [0.00, 0.70]	2014
Chang 2024	7	20	4	8	3.8%	0.54 [0.10, 2.84]	2024
Marra 2024	7	36	7	21		Not estimable	2024
**Subtotal (95% CI)**		**135**		**310**	**76.1%**	**0.25 [0.16, 0.40]**	
Total events	42		206				
Heterogeneity: Chi² = 2.47, df = 2 (P = 0.29); I² = 19%							
Test for overall effect: Z = 6.00 (P < 0.00001)							
**Total (95% CI)**		**241**		**417**	**100.0%**	**0.30 [0.21, 0.44]**	
Total events	58		235				
Heterogeneity: Chi² = 5.78, df = 4 (P = 0.22); I² = 31%							
Test for overall effect: Z = 6.21 (P < 0.00001)							
Test for subgroup differences: Chi² = 2.01, df = 1 (P = 0.16), I² = 50.2%							

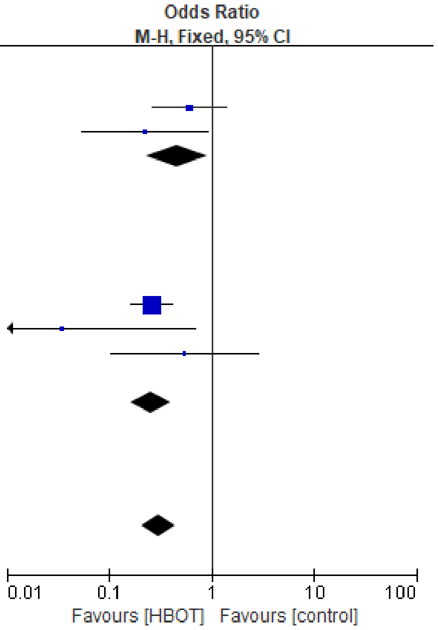

(G)

Study or Subgroup	HBOT Events	HBOT Total	Control Events	Control Total	Weight	Odds Ratio M-H, Fixed, 95% CI	Year
**19.1.1 RCT**							
Bouachour 1996	0	18	0	18		Not estimable	1996
Millar 2022	13	58	19	59	14.3%	0.61 [0.27, 1.39]	2022
Raj 2023	3	30	10	30	8.8%	0.22 [0.05, 0.91]	2023
**Subtotal (95% CI)**		**106**		**107**	**23.1%**	**0.46 [0.23, 0.93]**	
Total events	16		29				
Heterogeneity: Chi² = 1.46, df = 1 (P = 0.23); I² = 31%							
Test for overall effect: Z = 2.17 (P = 0.03)							
**19.1.2 Observational study**							
Roje 2008	35	99	196	289	63.2%	0.26 [0.16, 0.42]	2008
Yamada 2014	0	16	6	13	6.8%	0.03 [0.00, 0.70]	2014
Marra 2024	7	36	7	21	7.0%	0.48 [0.14, 1.65]	2024
Chang 2024	7	20	4	8		Not estimable	2024
**Subtotal (95% CI)**		**151**		**323**	**76.9%**	**0.26 [0.17, 0.40]**	
Total events	42		209				
Heterogeneity: Chi² = 2.69, df = 2 (P = 0.26); I² = 26%							
Test for overall effect: Z = 6.06 (P < 0.00001)							
**Total (95% CI)**		**257**		**430**	**100.0%**	**0.31 [0.21, 0.44]**	
Total events	58		238				
Heterogeneity: Chi² = 5.85, df = 4 (P = 0.21); I² = 32%							
Test for overall effect: Z = 6.28 (P < 0.00001)							
Test for subgroup differences: Chi² = 1.86, df = 1 (P = 0.17), I² = 46.1%							

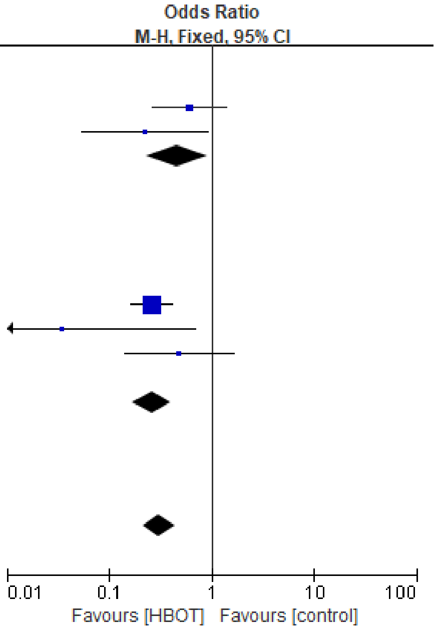

Supplementary Figure S4. Sensitivity analysis according to wound necrosis. Sensitivity analysis by omitting each study. (A) Omitting study by Bouachour et al., (B) Omitting study by Millar et al., (C) Omitting study by Roje et al., and (D) Omitting study by Marra et al..

(A)

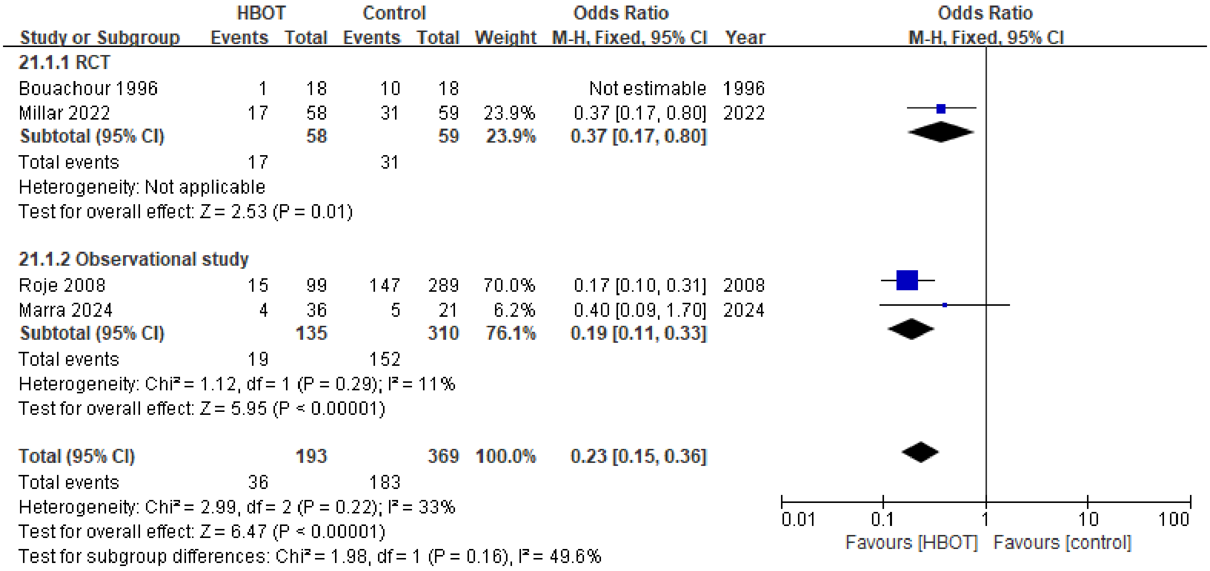

(B)

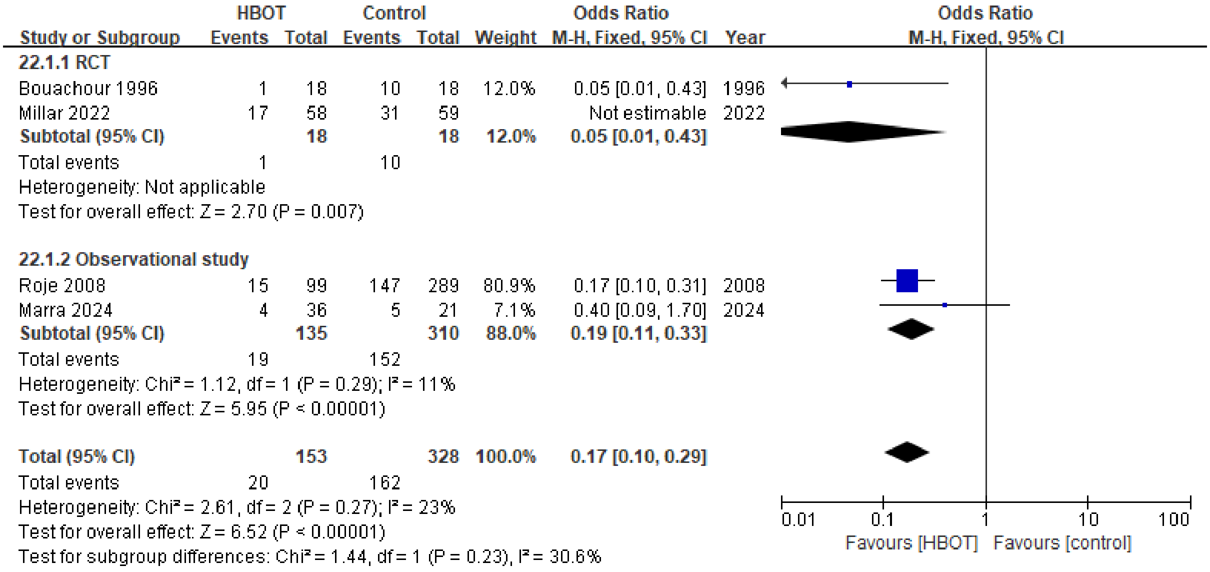

(C)

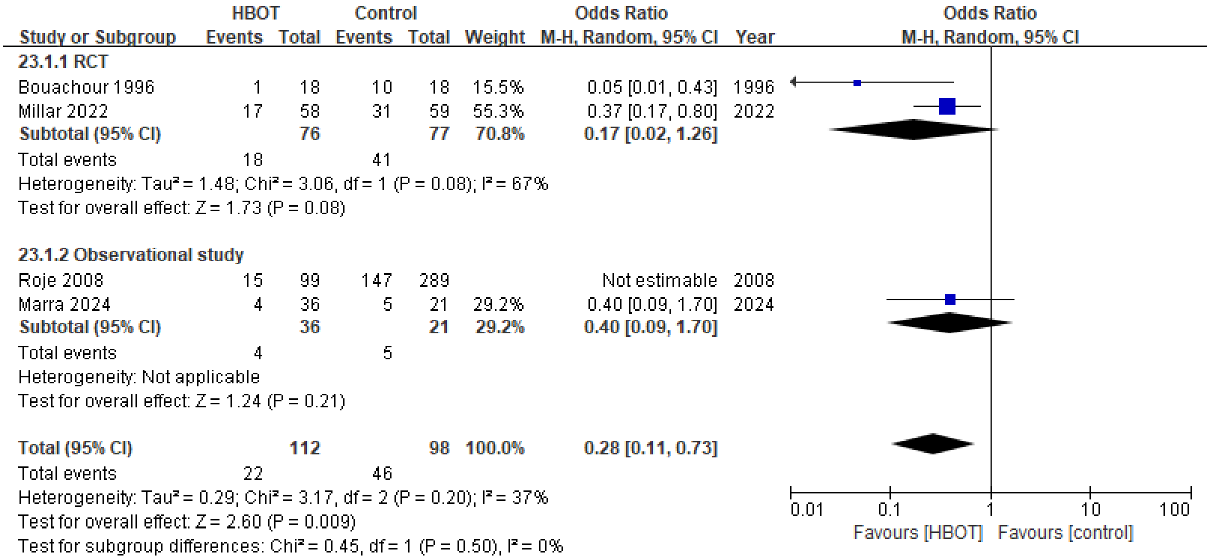
HBOT
Control
Odds Ratio
Odds Ratio
Study or Subgroup Events Total Events Total Weight M-H, Random, 95% CI Year
M-H, Random, 95% CI
23.1.1 RCT
Bouachour 1996 1 18 10 18 15.5% 0.05 [0.01, 0.43] 1996
Millar 2022 17 58 31 59 55.3% 0.37 [0.17, 0.80] 2022
Subtotal (95% CI) 76 77 70.8% 0.17 [0.02, 1.26]
Total events 18 41
Heterogeneity: Tau² = 1.48; Chi² = 3.06, df = 1 (P = 0.08); I² = 67%
Test for overall effect: Z = 1.73 (P = 0.08)
23.1.2 Observational study
Roje 2008 15 99 147 289 Not estimable 2008
Marra 2024 4 36 5 21 29.2% 0.40 [0.09, 1.70] 2024
Subtotal (95% CI) 36 21 29.2% 0.40 [0.09, 1.70]
Total events 4 5
Heterogeneity: Not applicable
Test for overall effect: Z = 1.24 (P = 0.21)
Total (95% CI) 112 98 100.0% 0.28 [0.11, 0.73]
Total events 22 46
Heterogeneity: Tau² = 0.29; Chi² = 3.17, df = 2 (P = 0.20); I² = 37%
Test for overall effect: Z = 2.60 (P = 0.009)
Test for subgroup differences: Chi² = 0.45, df = 1 (P = 0.50), I² = 0%
0.01 0.1 1 10 100
Favours [HBOT] Favours [control]

(D)

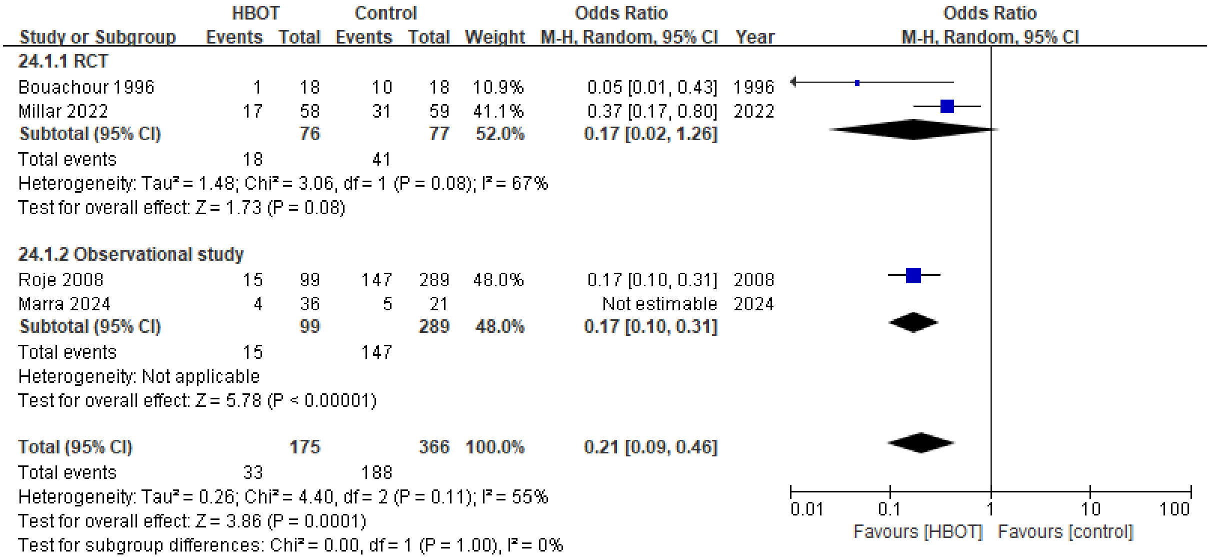
HBOT
Control
Odds Ratio
Odds Ratio
Study or Subgroup Events Total Events Total Weight M-H, Random, 95% CI Year
M-H, Random, 95% CI
24.1.1 RCT
Bouachour 1996 1 18 10 18 10.9% 0.05 [0.01, 0.43] 1996
Millar 2022 17 58 31 59 41.1% 0.37 [0.17, 0.80] 2022
Subtotal (95% CI) 76 77 52.0% 0.17 [0.02, 1.26]
Total events 18 41
Heterogeneity: Tau² = 1.48; Chi² = 3.06, df = 1 (P = 0.08); I² = 67%
Test for overall effect: Z = 1.73 (P = 0.08)
24.1.2 Observational study
Roje 2008 15 99 147 289 48.0% 0.17 [0.10, 0.31] 2008
Marra 2024 4 36 5 21 Not estimable 2024
Subtotal (95% CI) 99 289 48.0% 0.17 [0.10, 0.31]
Total events 15 147
Heterogeneity: Not applicable
Test for overall effect: Z = 5.78 (P < 0.00001)
Total (95% CI) 175 366 100.0% 0.21 [0.09, 0.46]
Total events 33 188
Heterogeneity: Tau² = 0.26; Chi² = 4.40, df = 2 (P = 0.11); I² = 55%
Test for overall effect: Z = 3.86 (P = 0.0001)
Test for subgroup differences: Chi² = 0.00, df = 1 (P = 1.00), I² = 0%
0.01 0.1 1 10 100
Favours [HBOT] Favours [control]

Supplementary Figure S5. Sensitivity analyses using the smaller-wound subgroup (≤50 cm^2) from Chang et al. (A) Time to wound healing. (B) Wound infection. In the primary analyses, the >50 cm^2 subgroup from Chang et al. was used because it was considered more representative of severe traumatic limb injury. In these sensitivity analyses, the ≤50 cm^2 subgroup was substituted while all other included studies remained unchanged. The two wound-size subgroups were not included simultaneously in the same meta-analysis.

(A)

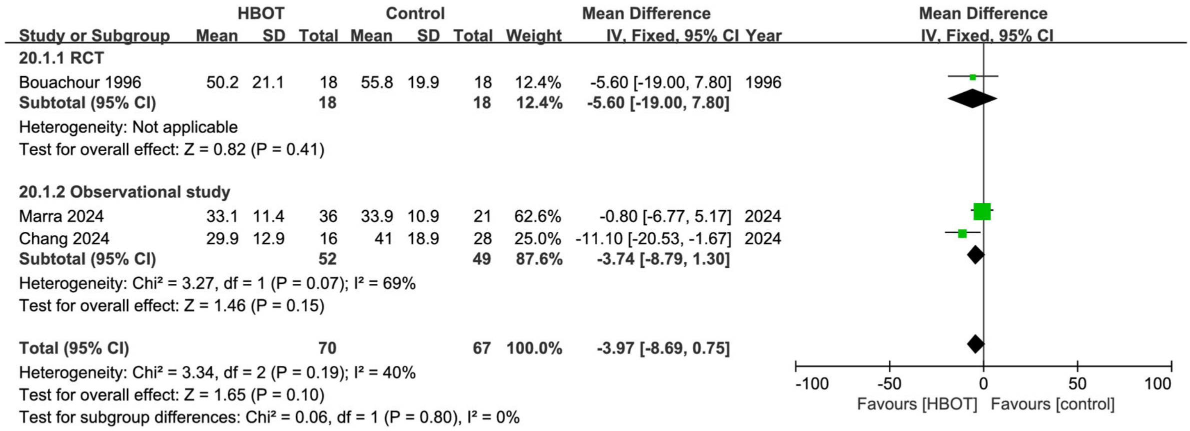

(B)

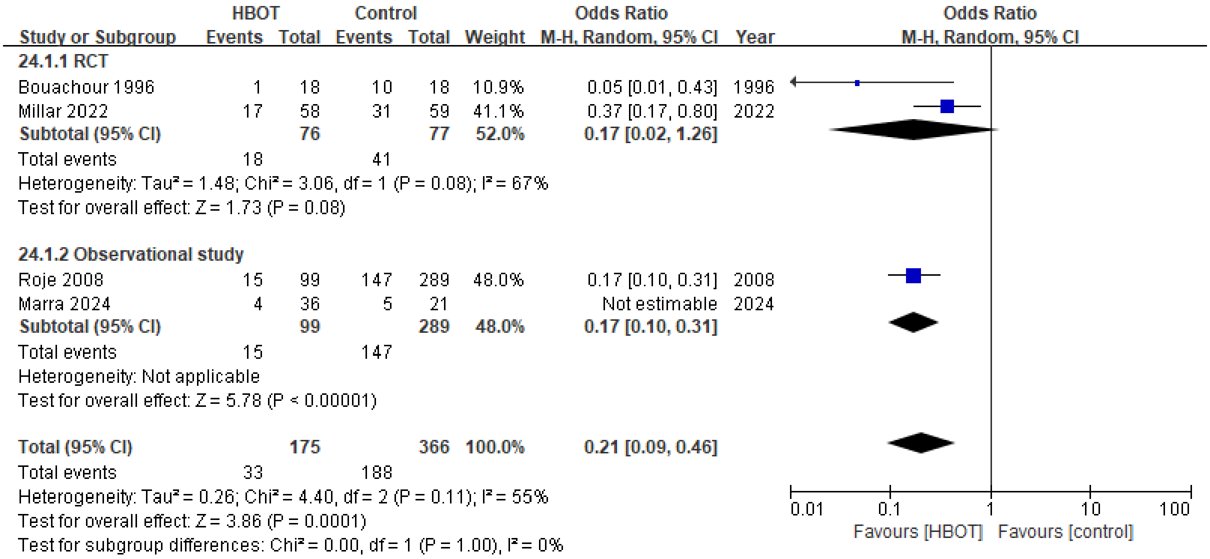

Supplement: Supplementary file 1 [file medicina-62-01589-s001.zip › medicina-4408833-supplementary.pdf]
